# Validation of miRNA prognostic power in hepatocellular carcinoma using expression data of independent datasets

**DOI:** 10.1038/s41598-018-27521-y

**Published:** 2018-06-15

**Authors:** Ádám Nagy, András Lánczky, Otília Menyhárt, Balázs Győrffy

**Affiliations:** 1 MTA TTK Lendület Cancer Biomarker Research Group, Institute of Enzymology, Magyar Tudósok körútja 2, 1117 Budapest, Hungary; 2 Semmelweis University 2nd Dept. of Pediatrics, Tűzoltó utca 7-9, 1094 Budapest, Hungary

## Abstract

Multiple studies suggested using different miRNAs as biomarkers for prognosis of hepatocellular carcinoma (HCC). We aimed to assemble a miRNA expression database from independent datasets to enable an independent validation of previously published prognostic biomarkers of HCC. A miRNA expression database was established by searching the TCGA (RNA-seq) and GEO (microarray) repositories to identify miRNA datasets with available expression and clinical data. A PubMed search was performed to identify prognostic miRNAs for HCC. We performed a uni- and multivariate Cox regression analysis to validate the prognostic significance of these miRNAs. The Limma R package was applied to compare the expression of miRNAs between tumor and normal tissues. We uncovered 214 publications containing 223 miRNAs identified as potential prognostic biomarkers for HCC. In the survival analysis, the expression levels of 55 and 84 miRNAs were significantly correlated with overall survival in RNA-seq and gene chip datasets, respectively. The most significant miRNAs were hsa-miR-149, hsa-miR-139, and hsa-miR-3677 in the RNA-seq and hsa-miR-146b-3p, hsa-miR-584, and hsa-miR-31 in the microarray dataset. Of the 223 miRNAs studied, the expression was significantly altered in 102 miRNAs in tumors compared to normal liver tissues. In summary, we set up an integrated miRNA expression database and validated prognostic miRNAs in HCC.

## Introduction

The incidence and mortality rates for liver cancer are increasing rapidly by approximately 3% per year^1^. Established risk factors include chronic infection with hepatitis B and/or hepatitis C viruses, obesity, diabetes, and alcohol consumption all of which contribute to the growing trend. Liver cancer incidence is high in East and South-East Asia and in Middle and Western Africa, while rates are low in South-Central and Western Asia and Northern and Eastern Europe^2^. Hepatocellular carcinoma (HCC) is the major histological type of liver cancer and accounts for approximately 80% of the total liver cancer burden worldwide^3^.

Hepatocellular carcinomas display notable clinical and biological heterogeneity^4^. HCC usually occurs with liver cirrhosis, characterized by accumulating genetically abnormal hepatocytes, which leads to cancer progression via unchecked cell proliferation and higher invasion and metastatic potential^5^. Using transcriptomic studies, further subgroups of HCC with distinct clinico-pathological features were also described^6,7^. Driver mutations involved in HCC progression were identified in the promoter of the telomerase reverse transcriptase (TERT) gene (in 60% of cases), in CTNNB1 (11–37%), which is part of the WNT pathway, in TP53 (10–30%), and in CDKN2A (2–12%), which has a role in the regulation of the cell cycle in G1 and G2 phases^8^.

MicroRNAs (miRNAs) are highly conserved small non-coding RNA molecules regulating the expression of approximately 30% of all human genes at the transcriptional and post-transcriptional levels^9^. Certain miRNAs have important roles in tumor development, progression, and in therapeutic response^10^. miRNAs serve as oncogenes or tumor suppressors in multiple cancers. In non-small cell lung cancer, low expression of hsa-miR-374a was correlated with poor survival outcome^11^. In colorectal cancer, the high expression of hsa-miR-185 and low expression of hsa-miR-133b were associated with metastasis and unfavorable clinical outcome^12^. hsa-miR-34a was linked to breast cancer^13^ and hsa-miR-10b to pancreatic cancer^14^. Derangement of the entire miRNA-ome was described in gastric cancer^15^.

Deregulation of miRNAs in HCC was demonstrated by a number of studies. The expression profile of miRNAs can serve as potential biomarkers for diagnosis, metastasis and invasion^16^, for prediction of therapeutic response, recurrence^10^, and of overall survival^17^ of HCC. Circulating miRNAs, including hsa-miR-939, hsa-miR-595, hsa-miR-519d, and hsa-miR-494, are capable of recognizing HCC in cirrhotic patients^18^.

In the future, miRNAs could be therapeutically utilized in anticancer therapy. The multi-kinase inhibitor sorafenib blocks the activity of VEGFR, PDGFR, and RAF and is included in standard care for advanced HCC. A phase 3 clinical study showed longer median survival and time to radiological progression for sorafenib treated patients than for untreated patients^19^. Conversely, sorafenib treatment can be influenced by the modulation of miRNAs - increasing expression of hsa-miR-122 resulted in sorafenib sensitivity^20^ and down-regulation of hsa-miR-34a resulted in sorafenib resistance in HCC cell lines^21^.

miRNA antagonists (antagomirs) could be used to inhibit the activity of cancer associated miRNAs (oncomirs). Antagomirs are chemically modified locked RNA-like nucleic acids that harbor various modifications for RNase protection^22^. HCC cell lines exhibit better response to chemotherapy when transfected with antisense-miR-21 during an IFN-α/5-fluorouracil (5-FU) combination therapy^23^. The suppression of hsa-miR-221 significantly decreased size and tumor number and increased the overall survival in a transgenic mouse model of liver cancer^24^. miRNA expression has also been found to have an important role in the treatment of HCC by interferons (IFNs). Higher expression of hsa-miR-146 resulted in IFN resistance^25^ and low expression of hsa-miR-26 resulted in IFN sensitivity in HCC cells^26^.

Although numerous miRNAs were suggested to have a prognostic power in HCC, in the literature, there is a scarcity of independent validation for these miRNAs. In this study, we first aimed to establish a list of previously published miRNAs with prognostic power, and then we intended to cross-validate these in an independent cohort of patients. For this, we assembled an HCC miRNA expression database using multiple, non-overlapping datasets, and then performed survival analysis to assess the prognostic potential for each gene. We also compared expression in liver cancer and normal samples to evaluate the change of the gene expression related to tumorigenesis.

## Results

### HCC miRNA gene expression database

Four HCC datasets met our criteria – GSE31384^17^, GSE10694^27^, GSE6857^28^, and TCGA. Among the GEO datasets, GSE6857 included 241 tumor samples, of which 240 samples also had non-cancerous pairs, GSE10694 had 78 cancer and corresponding non-cancerous samples, while GSE31384 contained cancer samples only. The TCGA database contained 372 primary tumor samples, of which 49 had a corresponding non-tumor pairs. There were no metastatic samples in the TCGA dataset. A summary of the datasets is given in Table 1.

**Table 1:** Summary of the datasets used for cross-validation in our study.

Study	Platform	Technology	Country	Data normalization	miRNA in platform	Sample #	Cancer #	Normal #
GSE10694	GPL6542	Microarray	China	Quantile normalization	119	156	78	78
GSE31384	GPL14140	Microarray	China	Quantile normalization	525	166	166	0
TCGA	Illumina	miRNA-Seq	USA	Reads per million miRNA mapped data	412	421	372	49
GSE6857	GPL4700	Microarray	USA	Median normalization	209	481	241	240
∑					845 unique miRNAs	1,224	857	367

Two different survival data were published – length of overall survival and disease free survival. Overall survival (OS) data were available for 372 patients in TCGA, 166 patients in GSE31384, and 77 patients in the GSE10694 dataset. The median follow up time of the entire database was 20.2 months (19.6, 34.0, and 15.8 months for TCGA, GSE31384, and GSE10694 datasets, respectively). Disease free survival (DFS) time was also available for 166 patients in the GSE31384 dataset with a median follow up of 17.5 months. However, only 54 patients had an event in GSE10694, and because of this small sample size we excluded GSE10694 from the survival analysis.

**Figure 1:**
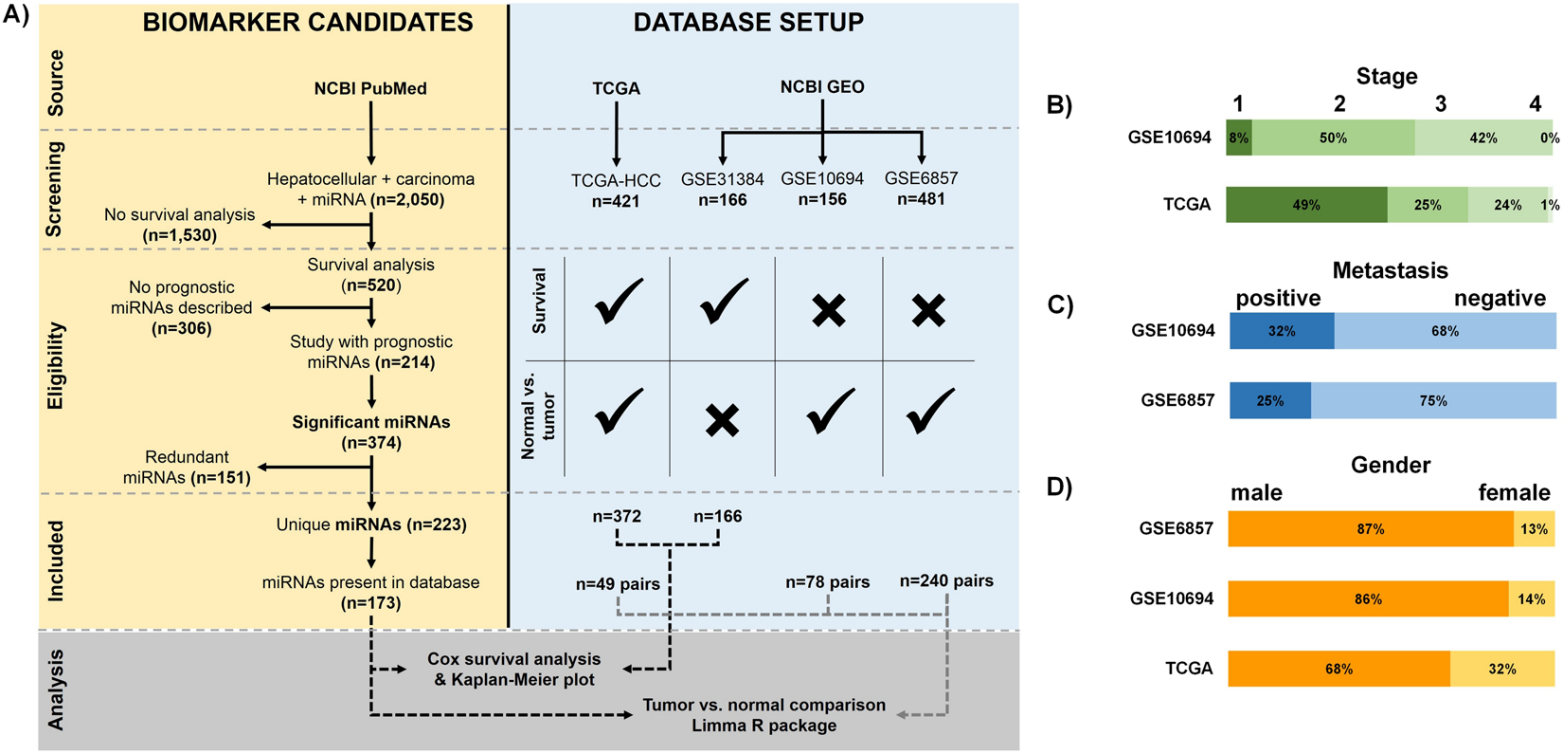
Overview of the statistical pipeline and sample numbers in the included datasets (**A**). Pathological characteristics of datasets including stage (**B**), metastasis (**C**), and gender (**D**).

Stage was available for TCGA and GSE10694, metastasis status for GSE6857 and GSE10694, and gender for TCGA, GSE6857, and GSE10694. An overview of the clinical parameters is presented in Fig. 1B–D. When we performed a survival analysis to inspect the correlation between the clinico-pathological parameters and survival, there was a significant correlation between OS and stage (*p* = 3.01E-06) in TCGA.

The GSE31384 dataset was different when compared to the other datasets. The labeled miRNAs from paired hepatocellular carcinoma and noncancerous liver were mixed and hybridized to the same array. Therefore, for these samples, the normalized log_2_ ratio (pCp-DY647/pCp-DY547) of the miRNA expression values represents the ratio of hepatocellular carcinoma/matched noncancerous liver tissue^17^.

**Figure 2:**
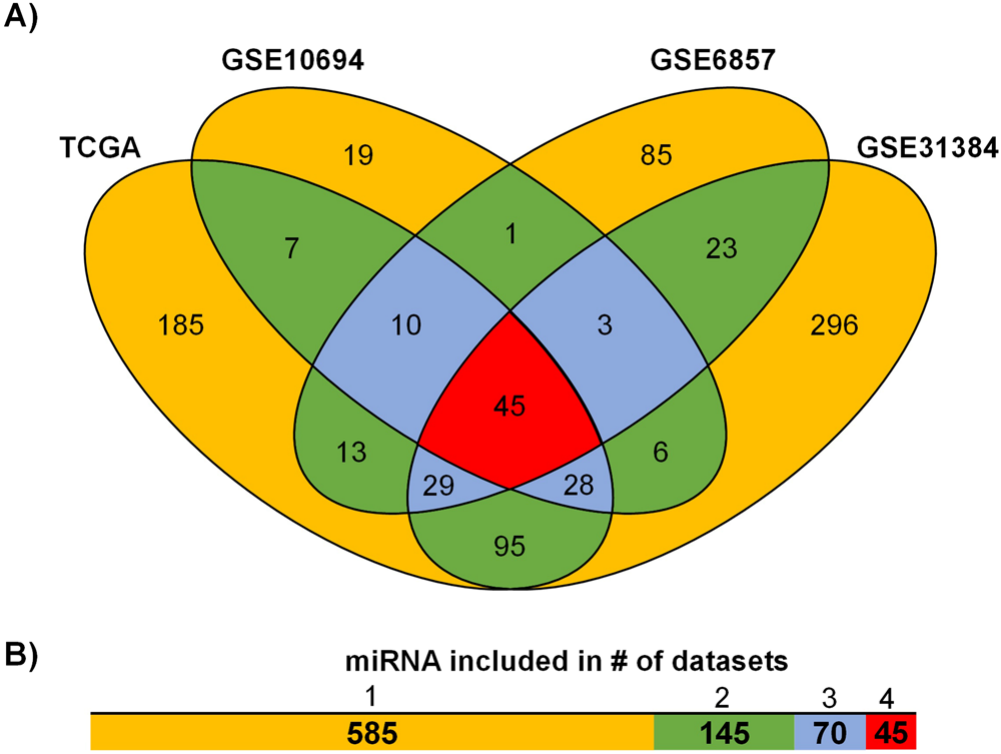
Slightly over 5% of all miRNAs were measured in each study. Overlap of detected miRNAs in each dataset (**A**) and aggregate summary (**B**).

Surprisingly, only a small proportion of miRNAs were measured in each platform (Fig. 2A). The entire database includes 845 unique miRNAs, of which 585 miRNAs were measured on one platform only, and 45 miRNAs were measured on each platform (Fig. 2B). The gene expression database combined with sample annotation and survival data is available in Supplemental Table 1.

### Literature-based list of prognostic miRNAs

The PubMed search resulted in 2,050 records for ‘hepatocellular’ + ‘carcinoma’ + ‘miRNA’ search terms, of which 214 studies described miRNAs with prognostic power (Supplemental Table 2). These articles contained 374 miRNA biomarker candidates, of which 151 miRNAs represented redundant publications (e.g., multiple studies describing the same miRNA). Of the 223 unique miRNAs, 173 have been measured in at least one dataset of our database (Fig. 1A).

### miRNAs with significant prognostic power

**Figure 3:**
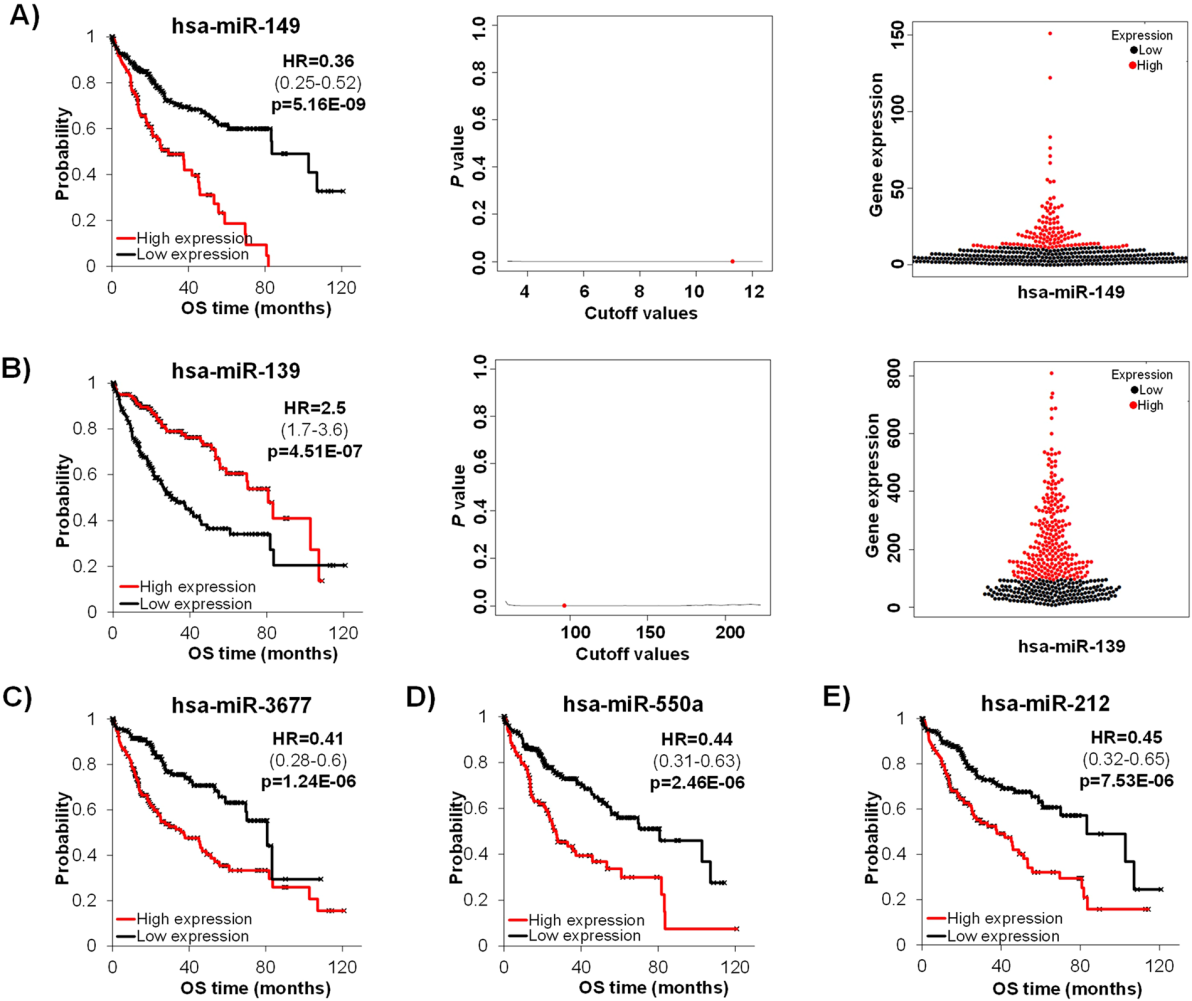
Top five best performing miRNAs based on expression in the tumor tissue using data of the TCGA dataset. Kaplan-Meier survival plots, p value vs. cutoff plots and beeswarm plots for hsa-miR-149 (**A**) and hsa-miR-139 (**B**) and Kaplan-Meier plots for hsa-miR-3677 (**C**), hsa-miR-550a (**D**), and hsa-miR-212 (**E**).

Of the 173 biomarker candidates, the expression of 55 miRNAs showed significant correlation with OS in the TCGA dataset (Supplemental Table 3A). The top five miRNAs included hsa-miR-149, hsa-miR-139, hsa-miR-3677, hsa-miR-550a, and hsa-miR-212 and are shown in Fig. 3A–E. For the top two miRNAs, hsa-miR-149 and hsa-miR-139, the *p* values achieved in the Cox regression across all possible cutoff values between the lower and upper quartiles of expression are also shown in Fig. 3A,B – it is important to note that the p values are highly significant irrespective of the used cutoff ***(****also see* Supplemental Fig. 1A–C *for cutoff vs. p value plots for the other genes)*. Beeswarm plots showed the expression distribution for these miRNAs (Fig. 3A,B and Supplemental Fig. 1D–F). In the multivariate analysis of OS, including the expression of all 55 significant miRNAs, stage and gender, 39 miRNAs remained significant (Supplemental Table 3A).

### Expression ratio of tumor vs. normal miRNAs as prognostic predictor

**Figure 4:**
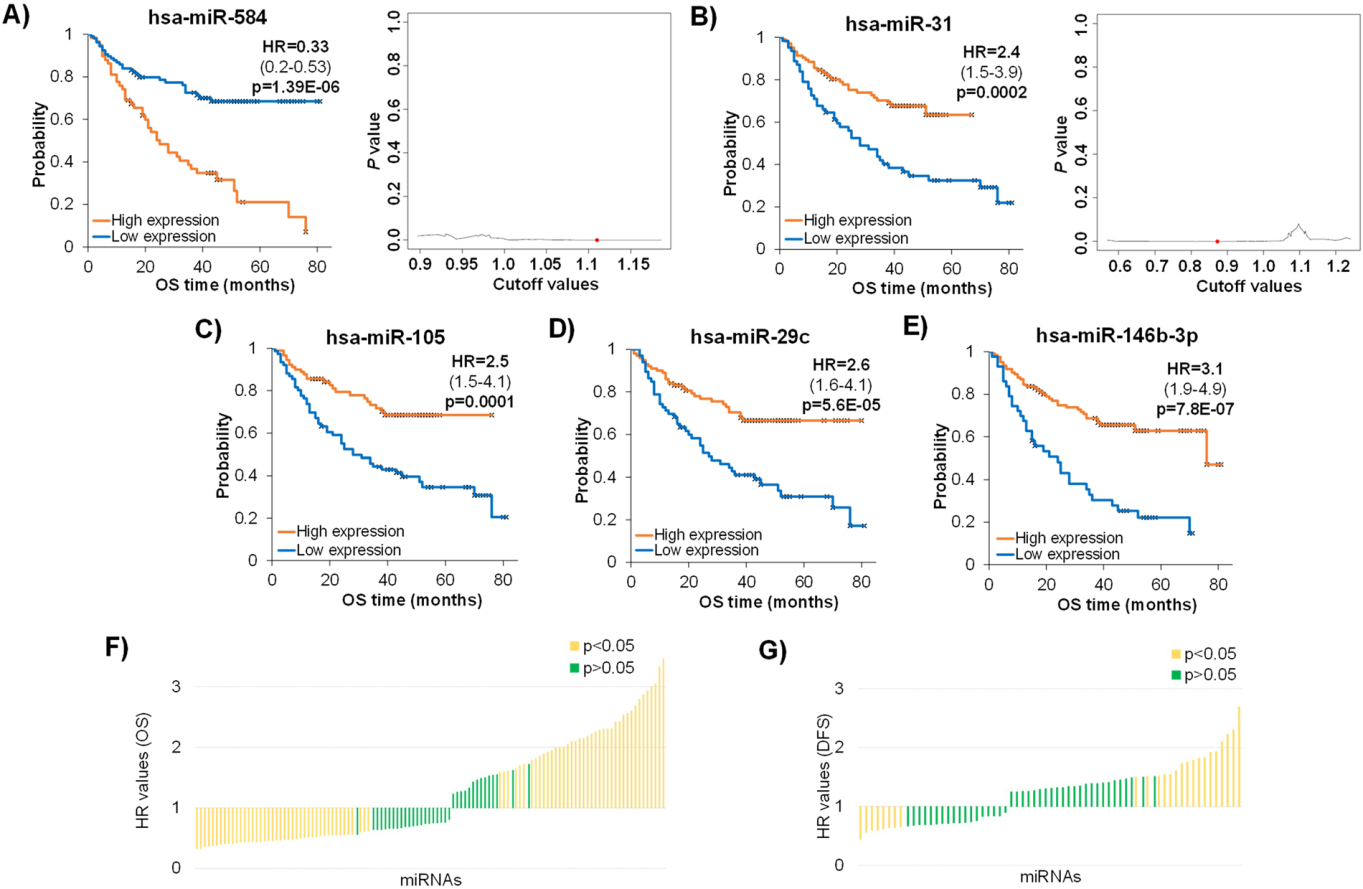
Best performing miRNAs when utilizing expression ratios of tumor and matched normal samples using data of the GSE31384 dataset. Kaplan-Meier survival plots and p value vs. cutoff plots for hsa-miR-584 (**A**) and hsa-miR-31 (**B**) and Kaplan-Meier plots for hsa-miR-105 (**C**), hsa-miR-29c (**D**), and hsa-miR-146b-3p (**E**). Ranked HR values for all investigated miRNAs for overall survival (**F**) and disease-free survival (**G**).

We also examined the prognostic value of the 173 miRNAs in the GSE31384 dataset, where the expression values of miRNAs represented ratios of the hepatocellular carcinoma vs. matched noncancerous liver tissue expression. As both OS and DFS data were available for this dataset, each miRNA was analyzed in the same survival setting as originally described. Uni- and multivariate survival analysis results for miRNAs with significant association to survival are listed in Supplemental Table 3B,C. The best performing five miRNAs included hsa-miR-584, hsa-miR-31, hsa-miR-146b-3p, hsa-miR-105, and hsa-miR-29c (Fig. 4A–E and Supplemental Fig. 2A–C). In addition, 25 miRNAs showed significant correlation with DFS, of which hsa-miR-126 (HR = 2.69, p = 4.90E-05), hsa-miR-122 (HR = 2.3, p = 5.21E-05), hsa-miR-106b (HR = 2.23, p = 5.56E-05), hsa-miR195 (HR = 1.93, p = 8.06E-04), and hsa-miR30a (HR = 1.92, p = 9.19E-04) had the highest prognostic power (Supplemental Table 3C). An ordered list of all miRNAs by HR-values using OS and DFS data is presented in Fig. 4F,G. Interestingly, less genes were significant in the DFS analysis than for OS. In the plot, an HR value below 1 corresponded to high expression associated with poor outcome, and values over 1 corresponded to high expression associated with good outcome in GSE31384. Of note, when comparing the TCGA and GSE31384, only two miRNAs were significant in both datasets after multivariate analysis, hsa-miR-10b and hsa-miR-195.

### miRNAs differentially expressed in HCC

**Figure 5:**
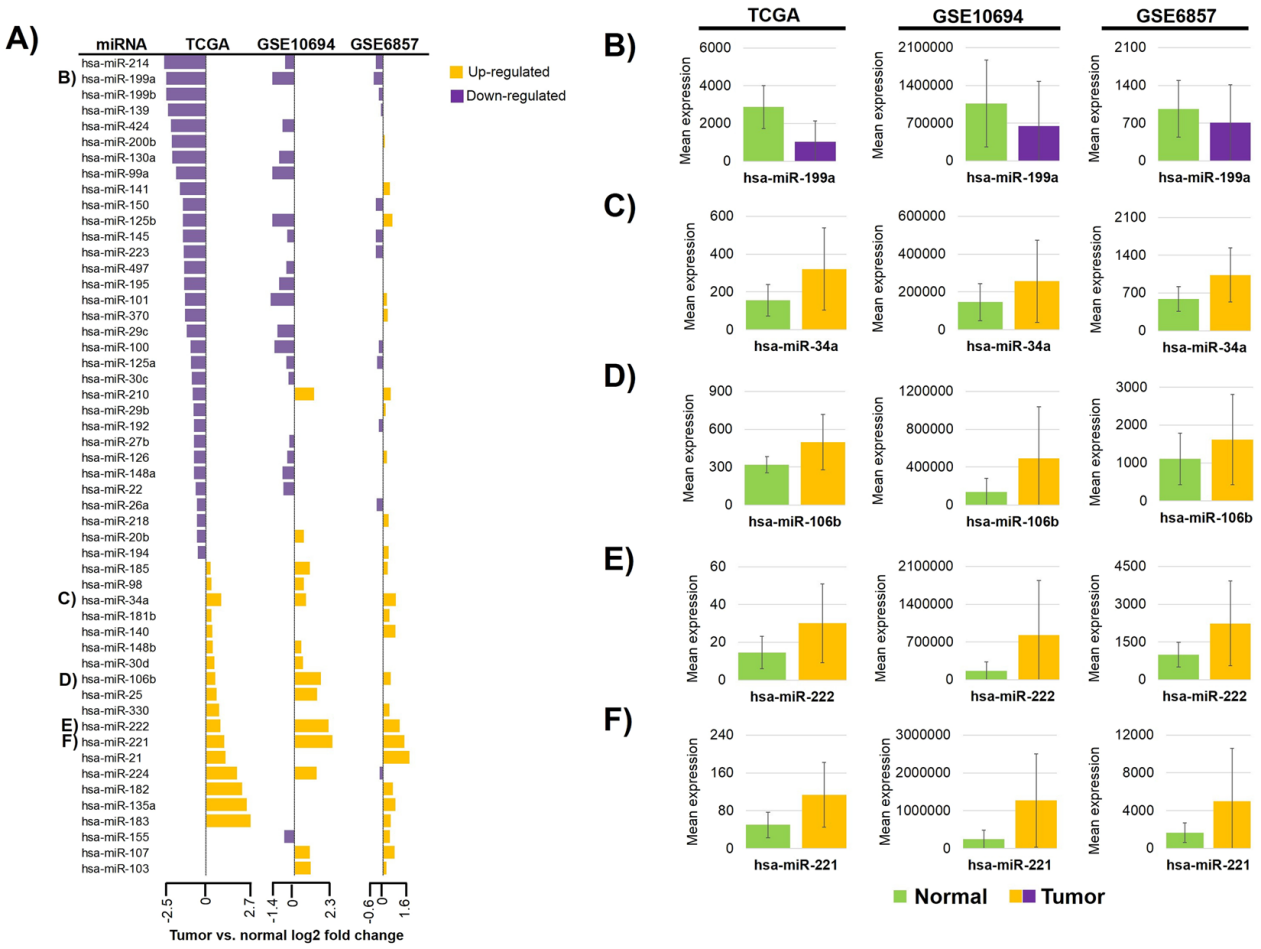
miRNAs significant in at least two studies (**A**) and miRNAs consistently down/up regulated in three datasets (**B**–**F**) – tumor vs. normal expression in TCGA, GSE10694 and GSE6857 datasets.

Of the 173 survival associated miRNAs, 113 had altered expression compared to normal tissues (Supplemental Table 4). Figure 5A shows the fold change values of miRNAs significantly changed in more than one dataset. Of the five miRNAs down-regulated in all three datasets, hsa-miR-199a showed the most significant expression change, also presented in Fig. 5B. Consistently up-regulated miRNAs included hsa-miR-34a, hsa-miR-106b, hsa-miR-222, and hsa-miR-221 (Fig. 5C–F).

### Correlation among different miRNAs

**Figure 6:**
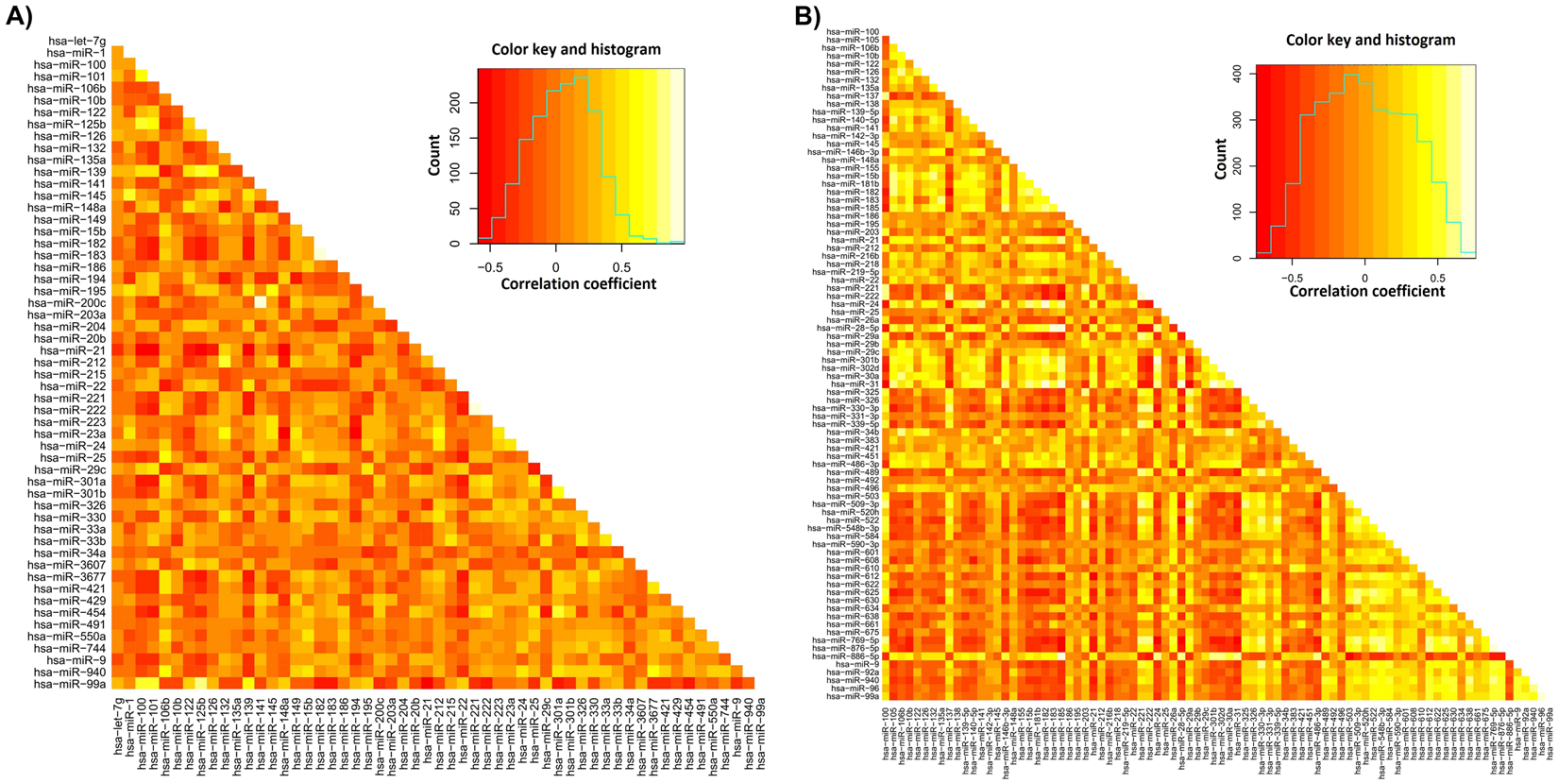
Heatmap showing correlation coefficients across all miRNAs significant in the survival analysis in the TCGA (**A**) and GSE31384 (**B**) datasets.

In order to assess the correlation among the miRNAs, we computed Spearman rank correlation for all miRNAs significant in the survival analysis. The miRNAs with the highest correlation in the TCGA dataset were hsa-miR-182/hsa-miR-183 (correlation coefficient = 0.98), hsa-miR-221/hsa-miR-222 (r = 0.91), and hsa-miR-200c/hsa-miR-141 (r = 0.9). Of these, for hsa-miR-221/hsa-miR-222, only hsa-miR-221 remained significant when running a multivariate analysis simultaneously including both miRNAs (p = 0.0386 and p = 0.357, respectively). For hsa-miR-182/hsa-miR-183 and hsa-miR-200c/hsa-miR-141, the correlation to survival was not significant in case we run them simultaneously in a multivariate analysis. In the GSE31384 dataset, the best-correlated miRNAs were hsa-miR-769-5p and hsa-miR-876-5p (r = 0.73). For these, the association between expression and OS was p = 0.000754/0.0023 in univariate and p = 0.0695/0.843 in multivariate analysis for hsa-miR-769-5p/hsa-miR-876-5p, respectively. These results suggest that many of the significant correlations can be explained by a correlation in gene expression. As our goal was to investigate each miRNA separately, we have not removed these from the analysis. We show a heatmap of correlation coefficients among all significant miRNAs in the TCGA and GSE31384 datasets in Fig. 6.

## Discussion

We assessed miRNAs for their prognostic power and started the analysis by performing a literature search to select miRNAs previously published as survival related in hepatocellular carcinoma. Simultaneously, we established a database integrating miRNA expression and clinical data derived from four independent hepatocellular carcinoma studies encompassing microarray and RNA-seq expression datasets. In this database, we validated the prognostic power of the miRNA biomarker candidates in a uni- and a multivariate analysis.

There were 55 miRNAs with expression that showed significant association with OS in HCC in the RNA-seq dataset. Among others, we found the strongest association with OS for hsa-miR-149, hsa-miR-139, hsa-miR-3677, hsa-miR-550a, and hsa-miR-212. Kaplan-Meier analysis showed that, with the exception of hsa-miR-139, increased expression of these miRNAs is associated with shorter overall survival.

A previous study revealed that hsa-miR-139 is a potential anti-oncogene in HCC^29^. Decreased expression of hsa-miR-139 was correlated with unfavorable survival outcome, invasion, microsatellite formation, and reduced differentiation. *In vitro* experiments overexpressing hsa-miR-139 in HCC cells reveled reduced migration and invasion capability of the cells and lower incidence of lung metastasis^29^. Hsa-miR-139 can interact with the ROCK2 kinase and reduce its expression in HCC cells, which is an important modulator of focal adhesion formation, cell motility and tumor cell invasion^29^. In another study, hsa-miR-139 inhibited T-cell factor-4 (TCF-4) protein expression via directly binding to the 3′UTR region of TCF-4 mRNA^30^. TCF-4 promotes initiation and progression of HCC by binding to β-catenin and transactivates Wnt target genes^31^. The decreased expression of hsa-miR-139 can induce tumor progression via the β-catenin/TCF-4 signaling pathway^30^. In this study, we confirmed these previous studies by linking low expression of hsa-miR-139 to poor clinical outcome.

Hsa-miR-550a is up-regulated in multiple cancers including HCC^32^. This miRNA regulates the invasion and migration of HCC cells via cytoplasmic polyadenylation element binding protein 4 (CPEB4). A previous study showed that hsa-miR-550a and CPEB4 expression were inversely associated in HCC samples and associated with unfavorable survival outcome^33^. This suggested, that the decreased expression of CPEB4 in HCC may be at least partially due to the upregulation of hsa-miR-550a. Our findings are consistent with these results, as we not only observed an increased expression of hsa-miR-550a in tumor tissue, but also a correlation to poor overall survival.

We also analyzed the prognostic efficiency of the previously reported miRNAs using a gene chip dataset (GSE31384). The expression values in this set represent the expression ratio of cancerous vs. noncancerous tissues. Among the 84 miRNAs, whose expression showed significant association with OS, the best performing miRNAs were hsa-miR-584, hsa-miR-31, hsa-miR-105, hsa-miR-29c, and hsa-miR-146b-3p. High levels of these miRNAs were associated with poor survival, except for hsa-miR-584. These observations coincide with a previous analysis that showed a significant correlation between the expression and overall survival of these best performing miRNAs^34^.

When comparing expression of HCC to normal liver tissue across three independent datasets, the top miRNAs that showed significant expression changes included hsa-miR-199a, hsa-miR-34a, hsa-miR-106b, hsa-miR-222, and hsa-miR-221. Hsa-miR-199a was down regulated in HCC tissues compared to adjacent non-tumor tissues by qRT-PCR analysis and *in vitro* experiments also showed that hsa-miR-199a was down regulated in HCC cell lines compared to a normal hepatocyte cell line^35^. Similar to these observations, we detected significantly decreased expression of hsa-miR-199a in HCC tissues. Hsa-miR-34a, a transcriptional target of TP53 in HCC^36^, was among the up-regulated miRNAs. *In vitro* studies showed that overexpression of hsa-miR-34a induced cell-cycle arrest and apoptosis by regulating cyclins, cyclin dependent kinases and apoptotic proteins^37^. Similarly, regarding our results, increased expression of hsa-miR-34a was measured in multiple studies of hepatocellular carcinoma^38,39^.

A few previous studies already analyzed miRNAs in the TCGA data for HCC and other tumor types. Andrés-Leon *et al*.^40^ identified several miRNAs as differentially expressed in multiple tumor types. They identified several up-regulated miRNAs in HCC including hsa-miR-421, hsa-miR-183, hsa-miR-182, hsa-miR-96 and hsa-miR-301, which were also overexpressed in our analysis. In addition, among the down-regulated miRNAs, hsa-miR-195, hsa-miR-139, hsa-miR-326 and hsa-miR-145 were also deregulated in our study. Jacobsen *et al*.^41^ described an analysis tool capable to examine recurrent miRNA-mRNA associations across several cancer types including glioblastoma multiforme, ovarian, colon and rectal adenocarcinoma, kidney renal clear cell carcinoma, breast, endometrioid, bladder, head and neck and lung adenocarcinoma. Unfortunately, liver hepatocellular carcinoma samples were not analyzed in their study. A survival analysis revealed 414 recurrent prognostic associations, where both gene and miRNA involved in each interaction delivered significant prognostic power in one or more cancer types^42^.

There are several limitations of our analysis. First, robust survival data were available for two datasets only, and only one of them (TCGA) had absolute expression values. The other dataset had only tumor/normal ratios, which combined two levels of data to evaluate the effect of expression changes on survival. A second major limitation is that approximately 5% of all miRNAs were measured in each of the four datasets and only 31% of miRNAs were available on more than two platforms – the non-overlapping investigation of miRNA-omes seems to be an issue also present for other tumor types^43^. This means that larger future studies are still needed to perform a comprehensive cross-validation of all potential miRNAs.

In summary, we constructed a miRNA gene expression database containing tumor and normal samples using four distinct hepatocellular carcinoma datasets. We used this integrated database to validate previously published biomarker candidates of HCC. The complete expression file combined with sample annotation and survival times is available as supplemental material. This database provides the option for future studies investigating miRNAs in HCC not only to perform an immediate independent validation of the results but also to rank investigated genes by comparing them to other candidate miRNAs.

## Methods

### Identifying gene chip based miRNA datasets

Microarray based gene expression series were downloaded from the NCBI Gene Expression Omnibus (GEO) (http://www.ncbi.nlm.nih.gov/geo/) repository. We used the search terms ‘hepatocellular’, ‘carcinoma’, ‘miRNA’ and ‘survival’ to identify miRNA expression datasets. We selected only those datasets that had available expression data and clinical survival information, and included at least 60 HCC patients. We acquired the expression of both the primary tumor samples and the corresponding normal samples in cases in which those were available. Due to different sensitivity, specificity and dynamic range of the utilized microarray platforms, each dataset was processed separately. We filtered to include only human miRNA genes and removed duplicated miRNAs. To be included, a miRNA had to be detectable in at least 75% of samples. We derived the mean expression of identical mature miRNAs (for example: hsa-miR-101-1 and hsa-miR-101-2 were combined). We transformed the logarithmic to a standard linear scale in case of the GSE10694 and GSE31384 datasets.

### Identifying RNA-seq based miRNA datasets

We uncovered one RNA-seq based HCC miRNA expression dataset generated by The Cancer Genome Atlas (TCGA) research network (http://cancergenome.nih.gov). In this, the pooled miRNA sequencing library was sequenced using Illumina technology. We acquired the preprocessed data normalized to reads per million mapped reads (RPM). Unmatched normal samples (n = 1) and recurrent samples (n = 3) were excluded from the analysis. The data filtering steps were identical to those used for the microarrays datasets.

### Literature survey of miRNAs prognostic in hepatocellular carcinoma

A literature search was performed using the keywords: ‘hepatocellular’, ‘carcinoma’, ‘miRNA’, and ‘survival’ in the NCBI PubMed database (https://www.ncbi.nlm.nih.gov/pubmed/) to identify miRNAs described in the literature as potential prognostic biomarkers for HCC. The literature search was performed on the 25^th^ of April 2017. Exclusion criteria included (a) studies not focused on miRNAs, (b) survival analysis not performed in studies, (c) data from animal or cell line studies, and (d) studies published in any language other than English^44^. Certain miRNAs were published in multiple studies – these were included only once in our analysis. Finally, we filtered the list of prognostic candidates to those that are in fact present in our combined HCC miRNA expression database.

### Survival analysis

The entire analysis for both microarray and RNA-seq datasets was performed in the R 2.15.0 statistical environment. Samples with missing survival data were excluded from the analysis. Hazard ratio (HR), 95% confidence intervals (CI) and log-rank *p* values were calculated. We applied the “survival” R package v2.38 (http://CRAN.R-project.org/package=survival/) for Cox regression analysis and the “survplot” R package v0.0.7 (http://www.cbs.dtu.dk/~eklund/survplot/) for generating Kaplan-Meier plots. We determined each percentile of miRNA expression between the lower and upper quartiles of expression as a cutoff point to divide patients into high and low expression groups as described previously^45^. Because of the low sample number, a cutoff outside the lower or upper quartile of expression could result in unreliable results. After this, the Cox regression analysis was performed separately for each cutoff. We used the cutoff with the lowest p value as the final cutoff in the Kaplan-Meier analysis. The cutoff values vs. p-values plot was generated to display the overall performance of the selected miRNAs.

In addition, a multivariate survival analysis was performed for the TCGA dataset including – in addition to the miRNAs – clinical data of stage and gender. To plot the distribution of miRNA expression in the high and low expression groups, we applied a one-dimensional scatter plot using the “beeswarm” R package (http://www.cbs.dtu.dk/~eklund/beeswarm/). Correlation between different miRNAs was assessed by computing Spearman rank correlations.

### Comparing miRNA expression between tumors and normal tissues

We applied the Limma R package to identify differentially expressed miRNAs between the HCC tumors and matched noncancerous tissues using the “model.matrix”, “lmFit”, “eBays” and “topTable” functions^46^. We also calculated the log2 fold change values (FC) for each miRNA by dividing the expression in the tumor samples with the measured expression in normal counterparts. The statistical significance threshold was set at *p* < 0.05. The setup of the database and the statistical computations are summarized in Fig. 1A.

### Data availability

The datasets analyzed during the current study are available in the Gene Expression Omnibus (http://www.ncbi.nlm.nih.gov/geo/) and The Cancer Genome Atlas (http://cancergenome.nih.gov) repositories.

## Electronic supplementary material

Supplemental Table 1.

Supplementary Information
