# Author Correction: Validation of miRNA prognostic power in hepatocellular carcinoma using expression data of independent datasets

**DOI:** 10.1038/s41598-018-29514-3

**Published:** 2018-07-26

**Authors:** Ádám Nagy, András Lánczky, Otília Menyhárt, Balázs Győrffy

**Affiliations:** 1 MTA TTK Lendület Cancer Biomarker Research Group, Institute of Enzymology, Magyar Tudósok körútja 2, 1117 Budapest, Hungary; 2 Semmelweis University 2nd Dept. of Pediatrics, Tűzoltó utca 7-9, 1094 Budapest, Hungary

Correction to: *Scientific Reports* 10.1038/s41598-018-27521-y, published online 15 June 2018

The Acknowledgements section in this Article is incomplete.

“The study was supported by the NVKP_16-1-2016-0037 and the FIEK_16-1-2016-0005 grants of the National Research, Development and Innovation Office, Hungary.”

should read:

“The study was supported by EFOP-3.6.3-VEKOP-16-2017-00009 grant and by the NVKP_16-1-2016-0037 and the FIEK_16-1-2016-0005 grants of the National Research, Development and Innovation Office, Hungary.”

