# Supplementary material for: Validation of miRNA prognostic power in hepatocellular carcinoma using expression data of independent datasets
Source: Sci Rep. 2018 Jun 15;8:9227. doi: 10.1038/s41598-018-27521-y (PMC6003936; doi:10.1038/s41598-018-27521-y)

Validation of miRNA prognostic power in hepatocellular carcinoma using expression data of independent datasets

Ádám Nagy[1,2], András Lánczky[1], Otília Menyhárt[1,2], Balázs Győrffy[1,2]

[1]*MTA TTK Lendület Cancer Biomarker Research Group, Institute of Enzymology, Magyar Tudósok körútja 2., 1117, Budapest, Hungary*
[2]*Semmelweis University 2nd Dept. of Pediatrics, Tűzoltó utca 7-9., 1094, Budapest, Hungary*

Corresponding author:

Balázs Győrffy MD PhD DSc
MTA TTK Lendület Cancer Biomarker Research Group, Institute of Enzymology,
Magyar Tudósok körútja 2., 1117, Budapest, Hungary
Tel: +3630-514-2822
Email: gyorffy.balazs@ttk.mta.hu

Supplemental Table 2. Complete list of previous studies evaluating prognostic miRNAs in hepatocellular carcinoma.

Abbreviations:

OS – Overall Survival
PFS – Progression-Free Survival
RFS – Recurrence/Relapse-Free Survival
MFS – Metastasis-Free Survival
TFS – Tumor-Free Survival
DFS – Disease-Free Survival
POS – Post-Operative Survival
TTR – Time To Recurrence
TTP – Time To Progression
DCR – DCR was defined as the percentage of patients who achieved complete response, partial response, or stable disease at first staging.
RR – Recurrence Rate

miRNAs	Survival	n	Prognostic association	*p*-Value	Reference
hsa-miR-301a<br>hsa-miR-132<br>hsa-miR-212<br>hsa-miR-489<br>hsa-miR-1468	OS	161	High expression/poor outcome	0.038137<br>0.006095<br>0.010822<br>0.011922<br>0.04855	1
hsa-miR-34a<br>hsa-miR-217	OS	-	High expression/better outcome	p<0.05	2
hsa-miR-9-3<br>hsa-miR-10b<br>hsa-miR-31<br>hsa-miR-519c<br>hsa-miR-522<br>hsa-miR-3660<br>hsa-miR-4784<br>hsa-miR-6883	OS	78	High expression/poor outcome	p<0.05	3
hsa-miR-4651	OS, RFS	111	OS & RFS: High expression/poor outcome	OS: 1.44E-04; RFS: 4.52E-03	4
hsa-miR-425-5p	OS, DFS	110	OS & DFS: High expression/poor outcome	OS: 0.001; DFS: 0.002	5
hsa-miR-138	OS	40	Hyper-methylation/poor outcome	0.03	6
hsa-miR-30b-5p	OS	90	High expression/better outcome	0.0116	7
hsa-miR-23b	OS	50	High expression/better outcome	p<0.01	8
hsa-miR-199a-5p	OS	56	High expression/better outcome	0.0056	9
hsa-miR-330	OS	78	High expression/poor outcome	0.0012	10
hsa-miR-590-3p	OS	60	High expression/better outcome	0.028	11
hsa-miR-9	OS, RFS	120	OS & RFS: High expression/better outcome	OS & RFS: p<0.001	12
hsa-miR-146b-5p	OS, DFS	60	OS & DFS: High expression/better outcome	OS: 0.018; DFS: 0.005	13
hsa-miR-105-1	OS, PFS	154	OS & PFS: High expression/better outcome	OS: 0.031; PFS: 0.038	14
hsa-miR-302d	OS	110	High expression/poor outcome	0.015	15
hsa-miR-34c-3p	OS	81	High expression/poor outcome	0.022	16
hsa-miR-10b-3p	OS	24	High expression/poor outcome	0.042	17
hsa-miR-181a-5p	OS	53<br>30	High expression/better outcome	0.0038<br>0.0037	18
hsa-mir-3677<br>hsa-mir-421<br>hsa-mir-326<br>hsa-mir-424<br>hsa-mir-511-2	OS	304	High expression/poor outcome	p<0.0001	19
hsa-miR-221	OS, DFS	135	OS & DFS: High expression/poor outcome	OS & DFS: p<0.001	20
hsa-miR-625<br>hsa-miR-550a<br>hsa-miR-520g<br>hsa-miR-494<br>hsa-miR-486-5p<br>hsa-miR-331-3p<br>hsa-miR-26a<br>hsa-miR-214<br>hsa-miR-200a<br>hsa-miR-199a-5p<br>hsa-miR-182<br>hsa-miR-141	OS<br>OS<br>OS, DFS<br>DFS<br>DFS<br>OS/DFS<br>OS/DFS<br>OS/DFS<br>OS<br>OS<br>DFS<br>OS	99<br>236<br>65<br>120<br>72<br>238<br>65<br>65<br>101<br>120<br>86<br>212	High expression/poor outcome	0.003<br>p<0.0010<br>OS & DFS: p<0.0010<br>0.0017<br>p<0.0500<br>OS:0.0030; DFS:0.0010<br>OS:0.0010; DFS:0.0250<br>OS:0.0280; DFS:<0.0010<br>p<0.0100<br>0.0002<br>0.039<br>0.002	21

hsa-miR-140-5p	OS/DFS	120		OS:0.0040; DFS:0.0030	
hsa-miR-137	OS	136		0.0110	
hsa-miR-135a	OS/DFS	50		OS:0.0200; DFS:0.0110	
hsa-miR-125a	OS	40		p<0.0010	
hsa-miR-124	DFS	131		p<0.0010	
hsa-miR-122	OS	217		0.004	
hsa-miR-101	DFS	197		p<0.0001	
hsa-miR-100	DFS	98		0.023	
hsa-let-7g	OS	40		0.024	
hsa-miR-122	OS	161	High expression/poor outcome	0.085	22
hsa-miR-33a	OS	-	High expression/better outcome	-	23
hsa-miR-638	OS, DFS	113	OS & DFS: High expression/better outcome	OS: 0.0003; DFS: 0.0001	24
hsa-miR-98-5p	OS	84	High expression/better outcome	0.031	25
hsa-miR-22	OS	372	High expression/better outcome	0.0441	26
hsa-miR-9-1			High expression/poor outcome	p<0.0001	
hsa-miR-9-2			High expression/poor outcome	p<0.0001	
hsa-miR-223			High expression/poor outcome	0.0421	
hsa-miR-139			High expression/better outcome	0.0099	
hsa-miR-33b			High expression/poor outcome	0.0070	
hsa-miR-21			High expression/poor outcome	0.0054	
hsa-miR-634	OS	31	High expression/better outcome	0.0002	27
hsa-miR-455	OS, DFS	104	OS & DFS: High expression/better outcome	OS & DFS: 0.0001	28
hsa-miR-885-5p	OS	51	High expression/better outcome	0.0021	29
hsa-miR-589-5p	OS, DFS	66	OS & DFS: High expression/better outcome	OS: 0.012; DFS: 0.004	30
hsa-miR-98	OS	144	High expression/better outcome	0.0125	31
hsa-miR-210	OS, TFS	212	OS & TFS: High expression/poor outcome	OS: 0.012; TFS:0.036	32
hsa-miR-27b	OS, DFS	90	OS & DFS: High expression/poor outcome	OS: 0.016; DFS: 0.020	33
hsa-miR-139-5p	OS	153	High expression/better outcome	p<0.001	34
hsa-miR-105			High expression/poor outcome	0.0022	
hsa-miR-142-3p			High expression/better outcome	0.0436	
hsa-miR-145*			High expression/better outcome	0.0001	
hsa-miR-181a*			High expression/better outcome	0.0405	
hsa-miR-15b			High expression/poor outcome	0.0051	
hsa-miR-181b			High expression/better outcome	0.0122	
hsa-miR-183			High expression/poor outcome	0.0118	
hsa-miR-186			High expression/poor outcome	0.025	
hsa-miR-18b*			High expression/better outcome	0.0385	
hsa-miR-26a			High expression/better outcome	0.0081	
hsa-miR-224			High expression/poor outcome	0.0152	
hsa-miR-23a*			High expression/better outcome	0.0368	
hsa-miR-29b			High expression/poor outcome	0.0202	
hsa-miR-29c			High expression/better outcome	0.0018	
hsa-miR-30a			High expression/better outcome	0.021	

hsa-miR-31			High expression/better outcome	0.0019	
hsa-miR-422a			High expression/better outcome	0.0048	
hsa-miR-496			High expression/poor outcome	0.0246	
hsa-miR-509-3p			High expression/poor outcome	0.0181	
hsa-miR-522			High expression/poor outcome	0.0021	
hsa-miR-548b-3p			High expression/poor outcome	0.0075	
hsa-miR-584			High expression/poor outcome	0.0041	
hsa-miR-601			High expression/poor outcome	0.015	
hsa-miR-675			High expression/poor outcome	0.0003	
hsa-miR-661			High expression/better outcome	0.0005	
hsa-miR-9*			High expression/poor outcome	0.0265	
hsa-miR-99b			High expression/better outcome	0.0412	
hsa-miR-200b			High expression/better outcome	0.0166	
hsa-miR-106b			High expression/poor outcome	0.0004	
hsa-miR-34b			High expression/better outcome	0.0019	
hsa-miR-301b			High expression/poor outcome	0.0111	
hsa-miR-28-5p			High expression/poor outcome	0.009	
hsa-miR-330-3p			High expression/poor outcome	0.0094	
hsa-miR-612			High expression/poor outcome	0.0128	
hsa-miR-769-5p			High expression/poor outcome	0.0028	
hsa-miR-146b-3p			High expression/better outcome	0.0038	
hsa-miR-367-3p	RFS	92	High expression/better outcome	0.0103	35
hsa-miR-15b	OS	156	High expression/poor outcome		36
hsa-miR-101	OS, RFS	78	OS & RFS: High expression/poor outcome	OS: 0.024; RFS: 0.019	37
hsa-miR-149	OS	145	High expression/better outcome	p<0.0001	38
hsa-miR-218	OS	156	High expression/better outcome	p<0.05	39
hsa-miR-130b	OS, DFS	85	OS & DFS: High expression/poor outcome	OS: 0.045; DFS: 0.003	40
hsa-miR-192-5p	OS, PFS	74	OS & PFS: High expression/poor outcome	OS & PFS: p<0.001	41
hsa-miR-29a-3p				OS: 0.001; PFS:0.023	
hsa-miR-29c	OS	91	High expression/better outcome	0.002	42
hsa-miR-1258	DFS	20	High expression/better outcome	0.0058	43
hsa-miR-33a-3p	OS, DFS	85	OS & DFS: High expression/better outcome	OS: 0.0335; DFS: 0.0295	44
hsa-miR-338-3p	MFS	163	High expression/better outcome	0.013	45
hsa-miR-129-2	OS, DFS	106	OS & DFS: High expression/better outcome	OS:0.0002; DFS:0.0006	46
hsa-miR-106b	OS, DFS	108	OS & DFS: High expression/poor outcome	OS & DFS: p<0.05	47
hsa-miR-155	OS, DFS	124	OS & DFS: High expression/poor outcome	OS: 0.0004; DFS: 0.0006	48
hsa-miR-28-5p	OS, TTR	228	OS & TTR: High expression/better outcome	OS: 0.003; TTR: 0.01	49
hsa-miR-630	OS, DFS	97	OS & DFS: High expression/better outcome	OS:0.0379; DFS:0.0072	50
hsa-miR-21	OS	244	High expression/poor outcome	0.00077	51
hsa-miR-3607			High expression/better outcome	0.00094	
hsa-miR-18a			High expression/poor outcome	0.0021	
hsa-miR-182			High expression/poor outcome	0.0019	
hsa-miR-221			High expression/poor outcome	0.0077	

hsa-miR-25			High expression/poor outcome	0.0006	
hsa-miR-148			High expression/better outcome	0.016	
hsa-miR-122	OS, DFS	62	OS & DFS: High expression/better outcome	OS: 0.011;<br>DFS: 0.008	52
hsa-miR-148a				OS: 0.033;<br>DFS: 0.027	
hsa-miR-192				OS & DFS: 0.021	
hsa-miR-215				OS: 0.050;<br>DFS: 0.035	
hsa-miR-1246				OS & DFS:<br>p<0.001	
hsa-miR-1290				OS: 0.013;<br>DFS: 0.011	
hsa-miR-122<br>hsa-miR-126<br>hsa-miR-30a<br>hsa-miR-22<br>hsa-miR-15a	RFS	180	High expression/better outcome	8.8E-11<br>9.6E-11<br>5E-12<br>2.8E-11<br>8.6E-14	53
hsa-miR-4782-3p	POS	27	High expression/better outcome	p<0.05	54
hsa-miR-148a<br>hsa-miR-148b<br>hsa-miR-152	OS	76	High expression/better outcome	p<0.001<br>0.107<br>0.012	55
hsa-miR-454	OS, DFS	265	OS & DFS: High expression/poor outcome	OS & DFS:<br>p<0.001	56
hsa-miR-519a	OS, DFS	116	OS & DFS: High expression/poor outcome	OS: 0.0002;<br>DFS: 0.0005	57
hsa-miR-140	RFS	Validation: 36<br>Screening: 18	High expression/poor outcome	Validation: 0.225;<br>Screening: 0.091	58
hsa-miR-214				Validation: 0.203;<br>Screening: 0.036	
hsa-miR-455				Validation: 0.362;<br>Screening: 0.013	
hsa-miR-3187			High expression/poor outcome	Validation: 0.061;<br>Screening: 0.013	
hsa-miR-608	OS, DFS	42	OS & DFS: High expression/better outcome	OS: 0.0388;<br>DFS: 0.0217	59
hsa-miR-100	RFS	140	High expression/better outcome	0.032	60
hsa-miR-339-5p	OS	100	High expression/better outcome	0.001	61
hsa-miR-497	OS, DFS	86	OS & DFS: High expression/better outcome	OS: 0.016;<br>DFS:0.020	62
hsa-miR-21	OS	31	High expression/poor outcome	0.001	63
hsa-miR-383	OS	64	High expression/better outcome	0.002	64
hsa-miR-375	DFS	Poorly diff. group: 17<br>High serum AFP group: 20<br>Clip score group 1~3: 25	High expression/better outcome	0.017<br>0.009<br>0.024	65
hsa-miR-192	OS	355<br>85	High expression/better outcome	0.0407<br>0.0289	66
hsa-miR-214<br>hsa-miR-199a<br>hsa-miR-199a*	OS	96	High expression/better outcome	p<0.001	67
hsa-miR-940	OS	46	High expression/better outcome	0.0371	68
hsa-miR-192<br>hsa-miR-204	OS	48	High expression/better outcome	0.013<br>0.017	69
hsa-miR423-5p	RFS	115<br>43<br>72	High expression/poor outcome	p<0.002<br>0.158<br>0.001	70

hsa-miR-149	OS	91	High expression/better outcome	0.0229	71
hsa-miR-122	OS, DFS	120	OS: High expression/poor outcome; DFS: High expression/better outcome	OS: 0.042; DFS: 0.711	72
hsa-miR-424	OS, DFS	95	OS & DFS: High expression/better outcome	OS: 0.018; DFS: 0.008	73
hsa-miR-195	OS	92	High expression/better outcome	0.003	74
hsa-miR-34a-5p	OS, PFS	114	OS & PFS: High expression/better outcome	OS: 0.016; PFS: 0.165	75
hsa-miR-92a	OS, RFS	106	OS & RFS: High expression/poor outcome	OS & RFS: p<0.001	76
hsa-miR-96	OS	49	High expression/poor outcome	p<0.05	77
hsa-miR-19b	DFS	81	High expression/better outcome	0.022	78
hsa-miR-194	OS, DFS	56	OS & DFS: High expression/better outcome	OS: 0.0252; DFS: 0.0479	79
hsa-miR-188-5p	OS, DFS	Training: 120 Validation: 72	OS & DFS: High expression/better outcome	OS: 0.002; DFS: 0.001 OS: 0.014; DFS: 0.027	80
hsa-miR-122	OS	44	High expression/better outcome	0.0258	81
hsa-miR-424	OS, DFS	Training & Validation: 96	OS & DFS: High expression/better outcome	Training: OS: 0.007; DFS: 0.003 Validation: OS: 0.009; DFS: 0.005	82
hsa-miR-7<br>hsa-miR-107<br>hsa-miR-21<br>hsa-miR-103	DFS	88	High expression/poor outcome	0.0316<br>0.0103<br>0.0082<br>0.0593	83
hsa-miR-21	1-year survival<br>3-year survival<br>5-year survival	206<br>140<br>108	High expression/poor outcome	0.0484<br>0.0012<br>0.0012	84
hsa-miR-30c<br>hsa-miR-203a	OS	242	High expression/better outcome	p<0.001	85
hsa-miR-99b	OS, DFS	104	OS &DFS: High expression/poor outcome	OS: p<0.001; DFS: 0.001	86
hsa-miR-182<br>hsa-miR-331-3p	POS	67	High expression/poor outcome	0.001<br>0.013	87
hsa-miR-212	OS, DFS	86	OS & DFS: High expression/better outcome	OS: 0.003; DFS: 0.002	88
hsa-miR-429	OS, RFS	207	OS & RFS: High expression/poor outcome	OS & RFS: p<0.001	89
hsa-miR-451	OS, DFS	97	OS & DFS: High expression/better outcome	OS & DFS: p<0.001	90
hsa-miR-150	OS	120	High expression/better outcome	p<0.0001	91
hsa-miR-325	OS, PFS	99	OS & PFS: High expression/better outcome	OS & PFS: p<0.05	92
hsa-miR-92a	OS	90	High expression/poor outcome	0.0017	93
hsa-miR-940	OS	23	High expression/better outcome	0.004	94
hsa-miR-183<br>hsa-miR-96<br>hsa-miR-182	DFS	81	High expression/poor outcome	0.0370	95
hsa-mir-326<br>hsa-mir-3677<br>hsa-mir-511-1<br>hsa-mir-511-2<br>hsa-mir-9-1<br>hsa-mir-9-2	OS	327	High expression/poor outcome	0<br>0<br>0.001<br>0.012<br>0.02<br>0.008	96
hsa-mir-30d			High expression/better outcome	0.008	

hsa-miR-744	OS, RFS	96	OS & RFS: High expression/better outcome	OS: 0.015; RFS: 0.006	97
hsa-miR-21	OS	112	High expression/poor outcome	0.022	98
hsa-miR-128-3p	DFS	72	High expression/better outcome	p<0.05	99
hsa-miR-122	OS	All: 122<br>Male: 68<br>Female: 54	High expression/better outcome	p<0.01<br>p<0.01<br>0.16	100
hsa-miR-212	OS, DFS	95	OS & DFS: High expression/better outcome	OS: 0.002; DFS: p<0.001	101
hsa-miR-200a	OS	101	High expression/better outcome	p<0.01	102
hsa-miR-372	OS	120	High expression/better outcome	0.004	103
hsa-miR-216b	OS, DFS	150	OS & DFS: High expression/better outcome	OS & DFS: p<0.0001	104
hsa-miR-425-3p	TTP, PFS	56	TTP & PFS: High expression/better outcome	TTP: 0.002; PFS: 0.0008	105
hsa-miR-625	OS	83	High expression/better outcome	0.003	106
hsa-miR-101	OS	163	High expression/better outcome	p<0.0001	107
hsa-miR-128-2	OS	77	High expression/poor outcome	0.018	108
hsa-miR-148a	OS, RFS	Whole: 297<br>Male: 253<br>Female: 44	OS & RFS: High expression/better outcome	OS: 0.04; RFS: 0.393<br>OS: 0.002; RFS: 0.063<br>OS: 0.613; RFS: 0.500	109
hsa-miR-365	OS	15	High expression/better outcome	p<0.001	110
hsa-miR-138	OS	180	High expression/better outcome	p<0.001	111
hsa-miR-200a	OS, RFS	115	OS & RFS: High expression/better outcome	OS: 0.037; RFS: 0.004	112
hsa-miR-148b	DFS	40	High expression/better outcome	p<0.05	113
hsa-miR-146a	MFS	53	High expression/better outcome	p<0.001	114
hsa-miR-520g	OS, DFS	130	OS & DFS: High expression/poor outcome	OS & DFS: p<0.001	115
hsa-miR-224	OS	182	High expression/poor outcome	0.007	116
hsa-miR-622	OS	56	High expression/better outcome	p<0.001	117
hsa-miR-486-5p<br>hsa-miR-422a<br>hsa-miR-125b<br>hsa-miR-139-5p	RFS	116	High expression/better outcome	0<br>0.003<br>0.002<br>0.206	118
hsa-miR-4458	OS	30	High expression/better outcome	p<0.05	119
hsa-miR-1	OS	40	High expression/better outcome	0.04	120
hsa-miR-99b	DCR	19	High expression/poor outcome	0.074	121
hsa-miR-140-3p<br>hsa-miR-100<br>hsa-miR-125<br>hsa-miR-22	OS		High expression/better outcome	0.015<br>0.023<br>0.026<br>0.046	
hsa-miR-20b	OS	76	High expression/poor outcome	0.01	122
hsa-miR-491	OS	60	High expression/better outcome	-	123
hsa-miR-9	OS	200	High expression/poor outcome	p<0.0001	124
hsa-miR-100	RFS	98	High expression/better outcome	0.023	125
hsa-miR-610	OS	76	High expression/better	p<0.05	126

			outcome		
hsa-miR-106b	OS	104	High expression/poor outcome	0.004	127
hsa-miR-21	OS, DFS	119	OS & DFS: High expression/poor outcome	OS: 0.007; DFS: 0.001	128
hsa-miR-199a	OS	40	High expression/better outcome	p<0.001	129
hsa-miR-200a<br>hsa-miR-21<br>hsa-miR-122<br>hsa-miR-224-5p	OS	136	High expression/poor outcome	0<br>0.026<br>0.041<br>0.025	130
hsa-miR-193b	OS	131	High expression/better outcome	p<0.001	131
hsa-miR-125b	OS, DFS	49	OS & DFS: High expression/better outcome	OS: 0.037; DFS: 0.128	132
hsa-miR-331-3p	OS, DFS	Training: 120 Validation: 108	OS & DFS: High expression/poor outcome	OS: 0.003; DFS: 0.001 OS:0.002; DFS: 0.001	133
hsa-miR-130a	OS	102	High expression/better outcome	0.007	134
hsa-miR-126	OS	74	High expression/better outcome	0.004	135
hsa-miR-218	OS, DFS	60	OS & DFS: High expression/better outcome	OS: 0.0248; DFS: 0.0413	136
hsa-miR-492	OS	28	High expression/poor outcome	p<0.001	137
hsa-miR-29a	OS	223	High expression/better outcome	p<0.02	138
hsa-miR-24-3p	OS, DFS	84	OS & DFS: High expression/poor outcome	OS:0.002; DFS: 0.007	139
hsa-miR-1246	DFS	38	High expression/poor outcome	0.143	140
hsa-miR-30a	DFS	63	High expression/better outcome	0.015	141
hsa-miR-130b	OS, DFS	97	OS & DFS: High expression/poor outcome	OS: 0.022; DFS: 0.012	142
hsa-miR-224	OS, DFS	130	OS & DFS: High expression/poor outcome	p<0.001	143
hsa-miR-210	OS, PFS	113	OS & PFS: High expression/poor outcome	OS: p<0.001; PFS: 0.093	144
hsa-miR-142-3p	DFS	43	High expression/better outcome	0.049	145
hsa-miR-137	OS	136	High expression/better outcome	0.009	146
hsa-miR-23a	OS, RFS	78	OS & RFS: High expression/poor outcome	OS: 0.026; RFS: 0.016	147
hsa-miR-148b	OS	156	High expression/better outcome	0.006	148
hsa-miR-148a	OS, RFS	59	OS & RFS: High expression/better outcome	OS: 0.005; RFS: 0.045	149
hsa-miR-26a	OS	36	High expression/better outcome	p<0.001	150
hsa-miR-99a	OS, TFS	152	OS & TFS: High expression/better outcome	OS: 0.006; TFS: 0.004	151
hsa-miR-139	OS	31	High expression/better outcome	0.023	152
hsa-miR-25	OS	131	High expression/poor outcome	0.0192	153
hsa-miR-141	OS	212	High expression/poor outcome	0.002	154
hsa-miR-200c<br>hsa-miR-141	DFS	90	High expression/better outcome	p<0.01	155
hsa-miR-34a	OS	30	High expression/better outcome	0.001	156
hsa-miR-221	OS, RFS	-	OS & RFS: High expression/poor outcome	OS: p<0.001; RFS: 0.010	157
hsa-miR-24	OS, RFS	207	OS & RFS: High expression/poor outcome	OS: 1.92E-12; RFS: 2E-10	158
hsa-let-7g	OS	40	High expression/better outcome	0.024	159
hsa-miR-370	OS	86	High expression/better	0.015	160

			outcome		
hsa-miR-1<br>hsa-miR-122	OS	195	High expression/better outcome	0.011<br>0.036	161
hsa-miR-100	OS	134	High expression/better outcome	0.0017	162
hsa-miR-200a	OS	120	High expression/better outcome	p<0.01	163
hsa-miR-214	DFS	65	High expression/better outcome	0.028	164
hsa-miR-503	OS	125	High expression/better outcome	p<0.01	165
hsa-miR-216a<br>hsa-miR-217	DFS	50	High expression/poor outcome	0.0002<br>0.015	166
hsa-miR-195	RFS	135	High expression/better outcome	0.004	167
hsa-miR-224	OS	46	High expression/poor outcome	p<0.05	168
hsa-miR-140-5p	OS, DFS	120	OS & DFS: High expression/better outcome	OS: 0.011;<br>DFS: 0.006	169
hsa-miR-26a	OS, TTR	130	OS & TTR: High expression/better outcome	OS: p<0.001;<br>TTR: 0.025	170
hsa-miR-222	OS	49	High expression/poor outcome	0.036	171
hsa-miR-185	OS, TTR	95	OS & TTR: High expression/better outcome	OS: 0.02;<br>TTR: p<0.0001	172
hsa-miR-126	OS, TTR	68	OS & TTR: High expression/better outcome	OS: 0.009;<br>TTR: 0.011	173
hsa-miR-1323	OS, RFS	42	OS & RFS: High expression/poor outcome	OS: 0.009;<br>RFS: 0.005	174
hsa-miR-17-5p	OS	96	High expression/poor outcome	0.003	175
hsa-miR-20a	OS, RFS	100	OS & RFS: High expression/better outcome	OS & RFS:<br>p<0.001	176
hsa-miR-21<br>hsa-miR-221	OS	60	High expression/poor outcome	0.036<br>0.03	177
hsa-miR-29b	OS	87	High expression/better outcome	0.008	178
hsa-miR-155	OS, RFS	100	OS & RFS: High expression/poor outcome	OS & RFS:<br>p<0.001	179
hsa-miR-372	OS, RFS	108	OS & RFS: High expression/poor outcome	OS: 0.001;<br>RFS: 0.006	180
hsa-miR-101	OS, DFS	130	OS & DFS: High expression/better outcome	OS: 0.006;<br>DFS: 0.01	181
hsa-miR-200c	OS, DFS	23	OS & DFS: High expression/better outcome	OS: 0.023;<br>DFS: 0.047	182
hsa-miR-10b	OS	34	High expression/poor outcome	0.001	183
hsa-miR-203	OS, RFS	66	OS & RFS: High expression/better outcome	OS: 0.014;<br>RFS: 0.016	184
hsa-miR-19a<br>hsa-miR-886-5p<br>hsa-miR-126<br>hsa-miR-223<br>hsa-miR-24	OS	105	High expression/better outcome	0.02<br>0.042<br>0.012<br>0.007<br>0.003	185
hsa-miR-147			High expression/poor outcome	p<0.001	
hsa-miR-145	DFS	73	High expression/better outcome	p<0.05	186
hsa-miR-17-5p	OS, DFS	120	OS & DFS: High expression/poor outcome	OS: 0.012;<br>DFS: 0.011	187
hsa-miR-517c	TTR	61	High expression/poor outcome	0.043	188
hsa-miR-515-3p<br>hsa-miR-520h<br>hsa-miR-520f<br>hsa-miR-520g	OS			0.004<br>0.008<br>0.01<br>0.046	
hsa-miR-219-5p	OS	83	High expression/better outcome	0.005	189
hsa-miR-135a	OS, DFS	50	OS & DFS: High expression/poor outcome	OS: 0.02;<br>DFS: 0.011	190
hsa-miR-155	OS, RFS	100	OS & RFS: High expression/poor outcome	OS & RFS:<br>p<0.001	191
hsa-miR-29a-5p	OS, TTR	Training:	OS & TTR: High	OS: 0.1327;	192

		106<br>Validation:<br>112<br>BCLC 0/A:<br>142<br>BCLC B: 76	expression/poor outcome	TTR: 0.0015<br>OS: 0.0079;<br>TTR: 0.0015<br>TTR: 0.003<br>TTR: 0.286	
hsa-miR-214	DFS	50	High expression/better outcome	p<0.0001	193
hsa-miR-15a<br>hsa-miR-486-3p<br>hsa-miR-381	RFS	216	High expression/better outcome	0.012<br>0.003<br>0.020	194
hsa-miR-30c<br>hsa-miR-155<br>hsa-miR-432<br>hsa-miR-15b<br>hsa-miR-30b	RFS	216	High expression/poor outcome	0.016<br>0.013<br>0.008<br>0.016<br>0.004	194
hsa-miR-29a<br>hsa-miR-486-3p<br>hsa-miR-876-5p	OS	216	High expression/better outcome	0.014<br>0.005<br>0.041	194
hsa-miR-199b	PFS	35	High expression/better outcome	0.031	195
hsa-miR-99a	DFS	142	High expression/better outcome	0.011	196
hsa-miR-221	MFS	111	High expression/better outcome	0.006	197
hsa-miR-221	OS	46	High expression/poor outcome	p<0.05	198
hsa-miR-199-3p	OS, TFS	142<br>152	OS & TFS: High expression/better outcome	OS: 0.004;<br>TFS: p<0.001<br>OS: 0.001;<br>TFS: p<0.001	199
hsa-miR-139	OS, DFS	20	OS & DFS: High expression/better outcome	OS: 0.013;<br>DFS: 0.042	200
hsa-miR-21	OS	30	High expression/poor outcome	p<0.05	201
hsa-miR-22	DFS	160	High expression/better outcome	0.025	202
hsa-miR-122	OS	180	High expression/better outcome	2.3E-04	203
hsa-let-7g	OS	22<br>33	High expression/better outcome	0.013<br>0.049	204
hsa-miR-29b	DFS	127	High expression/better outcome	0.002	205
hsa-miR-222	OS, DFS	76	OS & DFS: High expression/poor outcome	OS: 0.003;<br>DFS: 0.011	206
hsa-miR-26a	OS	59<br>40	High expression/better outcome	0.01<br>0.02	207
hsa-miR-122	OS	64	High expression/poor outcome	p<0.001	208
hsa-miR-221	OS, RR	OS: 45<br>RR: 46	OS & RR: High expression/poor outcome	OS: 0.5;<br>RR: 0.0009	209
hsa-miR-122	OS, TTR	45	OS & TTR: High expression/better outcome	OS: 0.3;<br>TTR: 0.05	210
hsa-miR-125b	POS	75	High expression/better outcome	0.043	211
hsa-miR-18b-5p	RFS	73	High expression/poor outcome	p<0.05	212
hsa-miR-335-5p	OS, TTP	125	OS & TTP: High expression/better outcome	OS & TTP: p<0.001	213
hsa-miR-100<br>hsa-miR-99a<br>hsa-miR-99b<br>hsa-miR-125b<br>hsa-miR-378<br>hsa-miR-129-5p<br>hsa-miR-125a-5p<br>hsa-miR-497<br>hsa-miR-22<br>hsa-miR-140-3p<br>hsa-miR-145	RFS	73	High expression/poor outcome<br>High expression/poor outcome<br>High expression/poor outcome<br>High expression/poor outcome<br>High expression/poor outcome<br>High expression/poor outcome<br>High expression/poor outcome<br>High expression/poor outcome<br>High expression/poor outcome<br>High expression/poor outcome<br>High expression/poor outcome	p<0.0001<br>0.0006<br>0.0013<br>0.0028<br>0.0043<br>0.0075<br>0.0089<br>0.0123<br>0.0141<br>0.0306<br>0.0341	214

hsa-miR-221	High expression/better outcome	0.0441
hsa-miR-195	High expression/poor outcome	0.0487

Supplemental references

1 Liu, G. *et al.* A five-miRNA expression signature predicts survival in hepatocellular carcinoma. *APMIS : acta pathologica, microbiologica, et immunologica Scandinavica*, doi:10.1111/apm.12697 (2017).

2 Tian, Y. W. *et al.* Decreased levels of miR-34a and miR-217 act as predictor biomarkers of aggressive progression and poor prognosis in hepatocellular carcinoma. *Minerva medica* **108**, 108-113, doi:10.23736/S0026-4806.16.04616-4 (2017).

3 Zhen, Y. *et al.* Several microRNAs could predict survival in patients with hepatitis B-related liver cancer. *Scientific reports* **7**, 45195, doi:10.1038/srep45195 (2017).

4 Wu, X. M. *et al.* Diagnostic and prognostic potential of serum microRNA-4651 for patients with hepatocellular carcinoma related to aflatoxin B1. *Oncotarget*, doi:10.18632/oncotarget.16027 (2017).

5 Fang, F. *et al.* MiR-425-5p promotes invasion and metastasis of hepatocellular carcinoma cells through SCAI-mediated dysregulation of multiple signaling pathways. *Oncotarget* **8**, 31745-31757, doi:10.18632/oncotarget.15958 (2017).

6 Anwar, S. L. *et al.* hsa-mir-183 is frequently methylated and related to poor survival in human hepatocellular carcinoma. *World journal of gastroenterology* **23**, 1568-1575, doi:10.3748/wjg.v23.i9.1568 (2017).

7 Qin, X., Chen, J., Wu, L. & Liu, Z. MiR-30b-5p acts as a tumor suppressor, repressing cell proliferation and cell cycle in human hepatocellular carcinoma. *Biomedicine & pharmacotherapy = Biomedecine & pharmacotherapie* **89**, 742-750, doi:10.1016/j.biopha.2017.02.062 (2017).

8 Cao, J. *et al.* microRNA-23b suppresses epithelial-mesenchymal transition (EMT) and metastasis in hepatocellular carcinoma via targeting Pyk2. *Biomedicine & pharmacotherapy = Biomedecine & pharmacotherapie* **89**, 642-650, doi:10.1016/j.biopha.2017.02.030 (2017).

9 Huang, G. H., Shan, H., Li, D., Zhou, B. & Pang, P. F. MiR-199a-5p suppresses tumorigenesis by targeting clathrin heavy chain in hepatocellular carcinoma. *Cell biochemistry and function* **35**, 98-104, doi:10.1002/cbf.3252 (2017).

10 Hu, X., Feng, Y., Sun, L., Qu, L. & Sun, C. Roles of microRNA-330 and Its Target Gene ING4 in the Development of Aggressive Phenotype in Hepatocellular Carcinoma Cells. *Digestive diseases and sciences* **62**, 715-722, doi:10.1007/s10620-016-4429-2 (2017).

11 Ge, X. & Gong, L. MiR-590-3p suppresses hepatocellular carcinoma growth by targeting TEAD1. *Tumour biology : the journal of the International Society for Oncodevelopmental Biology and Medicine* **39**, 1010428317695947, doi:10.1177/1010428317695947 (2017).

12 Liu, Y. *et al.* Loss of N-Acetylgalactosaminyltransferase-4 Orchestrates Oncogenic MicroRNA-9 in Hepatocellular Carcinoma. *The Journal of biological chemistry* **292**, 3186-3200, doi:10.1074/jbc.M116.751685 (2017).

13 Li, C. *et al.* Down-regulation of miR-146b-5p by long noncoding RNA MALAT1 in hepatocellular carcinoma promotes cancer growth and metastasis. *Oncotarget* **8**, 28683-28695, doi:10.18632/oncotarget.15640 (2017).

14 Ma, Y. S. *et al.* High expression of miR-105-1 positively correlates with clinical prognosis of hepatocellular carcinoma by targeting oncogene NCOA1. *Oncotarget* **8**, 11896-11905, doi:10.18632/oncotarget.14435 (2017).

15 Chen, Y. L., Xu, Q. P., Guo, F. & Guan, W. H. MicroRNA-302d downregulates TGFBR2 expression and promotes hepatocellular carcinoma growth and invasion. *Experimental and therapeutic medicine* **13**, 681-687, doi:10.3892/etm.2016.3970 (2017).

16 Xiao, C. Z. *et al.* MicroRNA-34c-3p promotes cell proliferation and invasion in hepatocellular carcinoma by regulation of NCKAP1 expression. *Journal of cancer research and clinical oncology* **143**, 263-273, doi:10.1007/s00432-016-2280-7 (2017).

17 Yoon, E. L. *et al.* An Explorative Analysis for the Role of Serum miR-10b-3p Levels in Predicting Response to Sorafenib in Patients with Advanced Hepatocellular Carcinoma. *Journal of Korean medical science* **32**, 212-220, doi:10.3346/jkms.2017.32.2.212 (2017).

18 Nishida, N. *et al.* MicroRNAs for the Prediction of Early Response to Sorafenib Treatment in Human Hepatocellular Carcinoma. *Liver cancer* **6**, 113-125, doi:10.1159/000449475 (2017).

19 Lu, M. *et al.* A novel microRNAs expression signature for hepatocellular carcinoma diagnosis and prognosis. *Oncotarget* **8**, 8775-8784, doi:10.18632/oncotarget.14452 (2017).
20 Chen, F., Li, X. F., Fu, D. S., Huang, J. G. & Yang, S. E. Clinical potential of miRNA-221 as a novel prognostic biomarker for hepatocellular carcinoma. *Cancer biomarkers : section A of Disease markers* **18**, 209-214, doi:10.3233/CBM-161671 (2017).
21 Wu, L. *et al.* MetastamiRs: A promising choice for antihepatocellular carcinoma nucleic acid drug development. *Hepatology research : the official journal of the Japan Society of Hepatology* **47**, 80-94, doi:10.1111/hepr.12737 (2017).
22 Kim, S. S. *et al.* Plasma micoRNA-122 as a predictive marker for treatment response following transarterial chemoembolization in patients with hepatocellular carcinoma. *Journal of gastroenterology and hepatology* **32**, 199-207, doi:10.1111/jgh.13448 (2017).
23 Hou, H. *et al.* miR-33a expression sensitizes Lgr5+ HCC-CSCs to doxorubicin via ABCA1. *Neoplasma* **64**, 81-91, doi:10.4149/neo_2017_110 (2017).
24 Zhang, Y., Zhang, D., Jiang, J. & Dong, L. Loss of miR-638 promotes invasion and epithelial-mesenchymal transition by targeting SOX2 in hepatocellular carcinoma. *Oncology reports* **37**, 323-332, doi:10.3892/or.2016.5273 (2017).
25 Jiang, T. *et al.* MicroRNA-98-5p inhibits cell proliferation and induces cell apoptosis in hepatocellular carcinoma via targeting IGF2BP1. *Oncology research*, doi:10.3727/096504016X14821952695683 (2016).
26 Chen, M. *et al.* miR-22 targets YWHAZ to inhibit metastasis of hepatocellular carcinoma and its down-regulation predicts a poor survival. *Oncotarget* **7**, 80751-80764, doi:10.18632/oncotarget.13037 (2016).
27 Zhang, C. Z., Cao, Y., Fu, J., Yun, J. P. & Zhang, M. F. miR-634 exhibits anti-tumor activities toward hepatocellular carcinoma via Rab1A and DHX33. *Molecular oncology* **10**, 1532-1541, doi:10.1016/j.molonc.2016.09.001 (2016).
28 Qin, L., Zhang, Y., Lin, J., Shentu, Y. & Xie, X. MicroRNA-455 regulates migration and invasion of human hepatocellular carcinoma by targeting Runx2. *Oncology reports* **36**, 3325-3332, doi:10.3892/or.2016.5139 (2016).
29 Zhang, Z. *et al.* miR-885-5p suppresses hepatocellular carcinoma metastasis and inhibits Wnt/beta-catenin signaling pathway. *Oncotarget* **7**, 75038-75051, doi:10.18632/oncotarget.12602 (2016).
30 Zhang, X. *et al.* miR-589-5p inhibits MAP3K8 and suppresses CD90+ cancer stem cells in hepatocellular carcinoma. *Journal of experimental & clinical cancer research : CR* **35**, 176, doi:10.1186/s13046-016-0452-6 (2016).
31 Zhou, W. *et al.* MicroRNA-98 acts as a tumor suppressor in hepatocellular carcinoma via targeting SALL4. *Oncotarget* **7**, 74059-74073, doi:10.18632/oncotarget.12190 (2016).
32 Yang, Y. *et al.* MicroRNA-210 promotes cancer angiogenesis by targeting fibroblast growth factor receptor-like 1 in hepatocellular carcinoma. *Oncology reports* **36**, 2553-2562, doi:10.3892/or.2016.5129 (2016).
33 Sun, X. F. *et al.* MicroRNA-27b exerts an oncogenic function by targeting Fbxw7 in human hepatocellular carcinoma. *Tumour biology : the journal of the International Society for Oncodevelopmental Biology and Medicine* **37**, 15325-15332, doi:10.1007/s13277-016-5444-9 (2016).
34 Wang, Z. *et al.* Reanalysis of microRNA expression profiles identifies novel biomarkers for hepatocellular carcinoma prognosis. *Tumour biology : the journal of the International Society for Oncodevelopmental Biology and Medicine* **37**, 14779-14787, doi:10.1007/s13277-016-5369-3 (2016).
35 Xu, J. *et al.* The miR-367-3p Increases Sorafenib Chemotherapy Efficacy to Suppress Hepatocellular Carcinoma Metastasis through Altering the Androgen Receptor Signals. *EBioMedicine* **12**, 55-67, doi:10.1016/j.ebiom.2016.07.013 (2016).
36 Ji, W. B., Liu, X., Luo, Y. & Zhang, W. Z. High expression of miR-15b predicts poor prognosis for hepatocellular carcinoma after curative hepatectomy. *Oncology reports* **36**, 1901-1908, doi:10.3892/or.2016.4982 (2016).

37 Lv, X., Li, J. & Yang, B. Clinical effects of miR-101 on prognosis of hepatocellular carcinoma and carcinogenic mechanism of anti-miR-101. *Oncology reports* **36**, 2184-2192, doi:10.3892/or.2016.4980 (2016).
38 Lin, L., Zhang, Y. D., Chen, Z. Y., Chen, Y. & Ren, C. P. The clinicopathological significance of miR-149 and PARP-2 in hepatocellular carcinoma and their roles in chemo/radiotherapy. *Tumour biology : the journal of the International Society for Oncodevelopmental Biology and Medicine* **37**, 12339-12346, doi:10.1007/s13277-016-5106-y (2016).
39 Yang, L., Xu, Q., Xie, H., Gu, G. & Jiang, J. Expression of serum miR-218 in hepatocellular carcinoma and its prognostic significance. *Clinical & translational oncology : official publication of the Federation of Spanish Oncology Societies and of the National Cancer Institute of Mexico* **18**, 841-847, doi:10.1007/s12094-015-1447-z (2016).
40 Chang, R. M., Xu, J. F., Fang, F., Yang, H. & Yang, L. Y. MicroRNA-130b promotes proliferation and EMT-induced metastasis via PTEN/p-AKT/HIF-1alpha signaling. *Tumour biology : the journal of the International Society for Oncodevelopmental Biology and Medicine* **37**, 10609-10619, doi:10.1007/s13277-016-4919-z (2016).
41 Zhu, H. T. *et al.* Serum microRNA profiles as prognostic biomarkers for HBV-positive hepatocellular carcinoma. *Oncotarget* **7**, 45637-45648, doi:10.18632/oncotarget.10082 (2016).
42 Dong, C. W., Wang, Y. X., Du, F. T., Ding, W. & Hu, S. Y. Low miR-29c expression is a prognostic marker in hepatocellular carcinoma. *Genetics and molecular research : GMR* **15**, doi:10.4238/gmr.15037316 (2016).
43 Hu, M. *et al.* Loss of miR-1258 contributes to carcinogenesis and progression of liver cancer through targeting CDC28 protein kinase regulatory subunit 1B. *Oncotarget* **7**, 43419-43431, doi:10.18632/oncotarget.9728 (2016).
44 Han, S. Y. *et al.* MicroRNA-33a-3p suppresses cell migration and invasion by directly targeting PBX3 in human hepatocellular carcinoma. *Oncotarget* **7**, 42461-42473, doi:10.18632/oncotarget.9886 (2016).
45 Chen, J. S. *et al.* miR-338-3p inhibits epithelial-mesenchymal transition and metastasis in hepatocellular carcinoma cells. *Oncotarget*, doi:10.18632/oncotarget.10138 (2016).
46 Liu, Z. *et al.* Methylation-mediated repression of microRNA-129-2 suppresses cell aggressiveness by inhibiting high mobility group box 1 in human hepatocellular carcinoma. *Oncotarget* **7**, 36909-36923, doi:10.18632/oncotarget.9377 (2016).
47 Yen, C. S., Su, Z. R., Lee, Y. P., Liu, I. T. & Yen, C. J. miR-106b promotes cancer progression in hepatitis B virus-associated hepatocellular carcinoma. *World journal of gastroenterology* **22**, 5183-5192, doi:10.3748/wjg.v22.i22.5183 (2016).
48 Zhang, L. *et al.* MicroRNA-155 promotes tumor growth of human hepatocellular carcinoma by targeting ARID2. *International journal of oncology* **48**, 2425-2434, doi:10.3892/ijo.2016.3465 (2016).
49 Zhou, S. L. *et al.* miR-28-5p-IL-34-macrophage feedback loop modulates hepatocellular carcinoma metastasis. *Hepatology* **63**, 1560-1575, doi:10.1002/hep.28445 (2016).
50 Chen, W. X. *et al.* MicroRNA-630 suppresses tumor metastasis through the TGF-beta- miR-630-Slug signaling pathway and correlates inversely with poor prognosis in hepatocellular carcinoma. *Oncotarget* **7**, 22674-22686, doi:10.18632/oncotarget.8047 (2016).
51 Tang, S. *et al.* Stratification of Digestive Cancers with Different Pathological Features and Survival Outcomes by MicroRNA Expression. *Scientific reports* **6**, 24466, doi:10.1038/srep24466 (2016).
52 Ng, K. T. *et al.* Early-phase circulating miRNAs predict tumor recurrence and survival of hepatocellular carcinoma patients after liver transplantation. *Oncotarget* **7**, 19824-19839, doi:10.18632/oncotarget.7627 (2016).
53 Xie, Q. Y., Almudevar, A., Whitney-Miller, C. L., Barry, C. T. & McCall, M. N. A microRNA biomarker of hepatocellular carcinoma recurrence following liver transplantation accounting for within-patient heterogeneity. *BMC medical genomics* **9**, 18, doi:10.1186/s12920-016-0179-4 (2016).
54 Bo, W. *et al.* The tumor suppressor role of miR-4782-3p in hepatocellular carcinoma. *Oncology reports* **35**, 2107-2112, doi:10.3892/or.2016.4568 (2016).

55 Wang, F. *et al.* Circulating miR-148/152 family as potential biomarkers in hepatocellular carcinoma. *Tumour biology : the journal of the International Society for Oncodevelopmental Biology and Medicine* **37**, 4945-4953, doi:10.1007/s13277-015-4340-z (2016).
56 Zhou, L., Qu, Y. M., Zhao, X. M. & Yue, Z. D. Involvement of miR-454 overexpression in the poor prognosis of hepatocellular carcinoma. *European review for medical and pharmacological sciences* **20**, 825-829 (2016).
57 Tu, K., Liu, Z., Yao, B., Han, S. & Yang, W. MicroRNA-519a promotes tumor growth by targeting PTEN/PI3K/AKT signaling in hepatocellular carcinoma. *International journal of oncology* **48**, 965-974, doi:10.3892/ijo.2015.3309 (2016).
58 Liese, J. *et al.* A possible role of microRNAs as predictive markers for the recurrence of hepatocellular carcinoma after liver transplantation. *Transplant international : official journal of the European Society for Organ Transplantation* **29**, 369-380, doi:10.1111/tri.12733 (2016).
59 Wang, K., Liang, Q., Wei, L., Zhang, W. & Zhu, P. MicroRNA-608 acts as a prognostic marker and inhibits the cell proliferation in hepatocellular carcinoma by macrophage migration inhibitory factor. *Tumour biology : the journal of the International Society for Oncodevelopmental Biology and Medicine* **37**, 3823-3830, doi:10.1007/s13277-015-4213-5 (2016).
60 Huang, Y. H. *et al.* HBV polymerase overexpression due to large core gene deletion enhances hepatoma cell growth by binding inhibition of microRNA-100. *Oncotarget* **7**, 9448-9461, doi:10.18632/oncotarget.7021 (2016).
61 Wang, Y. L., Chen, C. M., Wang, X. M. & Wang, L. Effects of miR-339-5p on invasion and prognosis of hepatocellular carcinoma. *Clinics and research in hepatology and gastroenterology* **40**, 51-56, doi:10.1016/j.clinre.2015.05.022 (2016).
62 Zhang, L., Yu, Z., Xian, Y. & Lin, X. microRNA-497 inhibits cell proliferation and induces apoptosis by targeting YAP1 in human hepatocellular carcinoma. *FEBS open bio* **6**, 155-164, doi:10.1002/2211-5463.12032 (2016).
63 Hu, L. *et al.* Long noncoding RNA GAS5 suppresses the migration and invasion of hepatocellular carcinoma cells via miR-21. *Tumour biology : the journal of the International Society for Oncodevelopmental Biology and Medicine* **37**, 2691-2702, doi:10.1007/s13277-015-4111-x (2016).
64 Chen, L. *et al.* miR-383 inhibits hepatocellular carcinoma cell proliferation via targeting APRIL. *Tumour biology : the journal of the International Society for Oncodevelopmental Biology and Medicine* **37**, 2497-2507, doi:10.1007/s13277-015-4071-1 (2016).
65 Zhou, N. *et al.* Low-level expression of microRNA-375 predicts poor prognosis in hepatocellular carcinoma. *Tumour biology : the journal of the International Society for Oncodevelopmental Biology and Medicine* **37**, 2145-2152, doi:10.1007/s13277-015-3841-0 (2016).
66 Lian, J. *et al.* miR-192, a prognostic indicator, targets the SLC39A6/SNAIL pathway to reduce tumor metastasis in human hepatocellular carcinoma. *Oncotarget* **7**, 2672-2683, doi:10.18632/oncotarget.6603 (2016).
67 Wang, P. *et al.* miR-214/199a/199a* cluster levels predict poor survival in hepatocellular carcinoma through interference with cell-cycle regulators. *Oncotarget* **7**, 929-945, doi:10.18632/oncotarget.6137 (2016).
68 Ding, D. *et al.* miR-940 Suppresses Tumor Cell Invasion and Migration via Regulation of CXCR2 in Hepatocellular Carcinoma. *BioMed research international* **2016**, 7618342, doi:10.1155/2016/7618342 (2016).
69 Ge, Y. *et al.* MiRNA-192 [corrected] and miRNA-204 Directly Suppress lncRNA HOTTIP and Interrupt GLS1-Mediated Glutaminolysis in Hepatocellular Carcinoma. *PLoS genetics* **11**, e1005726, doi:10.1371/journal.pgen.1005726 (2015).
70 Wu, L. M. *et al.* Oncogenic role of microRNA-423-5p in hepatocellular carcinoma. *Hepatobiliary & pancreatic diseases international : HBPD INT* **14**, 613-618 (2015).
71 Luo, G. *et al.* miR-149 represses metastasis of hepatocellular carcinoma by targeting actin-regulatory proteins PPM1F. *Oncotarget* **6**, 37808-37823, doi:10.18632/oncotarget.5676 (2015).

72 Cho, H. J. *et al.* High circulating microRNA-122 expression is a poor prognostic marker in patients with hepatitis B virus-related hepatocellular carcinoma who undergo radiofrequency ablation. *Clinical biochemistry* **48**, 1073-1078, doi:10.1016/j.clinbiochem.2015.06.019 (2015).
73 Yao, H., Liu, X., Chen, S., Xia, W. & Chen, X. Decreased expression of serum miR-424 correlates with poor prognosis of patients with hepatocellular carcinoma. *International journal of clinical and experimental pathology* **8**, 14830-14835 (2015).
74 Wang, M., Zhang, J., Tong, L., Ma, X. & Qiu, X. MiR-195 is a key negative regulator of hepatocellular carcinoma metastasis by targeting FGF2 and VEGFA. *International journal of clinical and experimental pathology* **8**, 14110-14120 (2015).
75 Li, X. Y. *et al.* MicroRNA-34a-5p enhances sensitivity to chemotherapy by targeting AXL in hepatocellular carcinoma MHCC-97L cells. *Oncology letters* **10**, 2691-2698, doi:10.3892/ol.2015.3654 (2015).
76 Yang, W. *et al.* MicroRNA-92a contributes to tumor growth of human hepatocellular carcinoma by targeting FBXW7. *Oncology reports* **34**, 2576-2584, doi:10.3892/or.2015.4210 (2015).
77 Chen, Y., Dong, X., Yu, D. & Wang, X. Serum miR-96 is a promising biomarker for hepatocellular carcinoma in patients with chronic hepatitis B virus infection. *International journal of clinical and experimental medicine* **8**, 18462-18468 (2015).
78 Hung, C. L., Yen, C. S., Tsai, H. W., Su, Y. C. & Yen, C. J. Upregulation of MicroRNA-19b predicts good prognosis in patients with hepatocellular carcinoma presenting with vascular invasion or multifocal disease. *BMC cancer* **15**, 665, doi:10.1186/s12885-015-1671-5 (2015).
79 Zhao, Y. *et al.* MicroRNA-194 acts as a prognostic marker and inhibits proliferation in hepatocellular carcinoma by targeting MAP4K4. *International journal of clinical and experimental pathology* **8**, 12446-12454 (2015).
80 Fang, F. *et al.* MicroRNA-188-5p suppresses tumor cell proliferation and metastasis by directly targeting FGF5 in hepatocellular carcinoma. *Journal of hepatology* **63**, 874-885, doi:10.1016/j.jhep.2015.05.008 (2015).
81 Xu, Q. *et al.* MicroRNA-122 affects cell aggressiveness and apoptosis by targeting PKM2 in human hepatocellular carcinoma. *Oncology reports* **34**, 2054-2064, doi:10.3892/or.2015.4175 (2015).
82 Yang, H. *et al.* MicroRNA-424 inhibits Akt3/E2F3 axis and tumor growth in hepatocellular carcinoma. *Oncotarget* **6**, 27736-27750, doi:10.18632/oncotarget.4811 (2015).
83 Chen, W. S. *et al.* miRNA-7/21/107 contribute to HBx-induced hepatocellular carcinoma progression through suppression of maspin. *Oncotarget* **6**, 25962-25974, doi:10.18632/oncotarget.4504 (2015).
84 Shi, K. Q. *et al.* Hepatocellular carcinoma associated microRNA expression signature: integrated bioinformatics analysis, experimental validation and clinical significance. *Oncotarget* **6**, 25093-25108, doi:10.18632/oncotarget.4437 (2015).
85 Liu, D. *et al.* Downregulation of miRNA-30c and miR-203a is associated with hepatitis C virus core protein-induced epithelial-mesenchymal transition in normal hepatocytes and hepatocellular carcinoma cells. *Biochemical and biophysical research communications* **464**, 1215-1221, doi:10.1016/j.bbrc.2015.07.107 (2015).
86 Yang, J., Liu, X., Yuan, X. & Wang, Z. miR-99b promotes metastasis of hepatocellular carcinoma through inhibition of claudin 11 expression and may serve as a prognostic marker. *Oncology reports* **34**, 1415-1423, doi:10.3892/or.2015.4104 (2015).
87 Chen, L., Chu, F., Cao, Y., Shao, J. & Wang, F. Serum miR-182 and miR-331-3p as diagnostic and prognostic markers in patients with hepatocellular carcinoma. *Tumour biology : the journal of the International Society for Oncodevelopmental Biology and Medicine* **36**, 7439-7447, doi:10.1007/s13277-015-3430-2 (2015).
88 Tu, H. *et al.* MicroRNA-212 inhibits hepatocellular carcinoma cell proliferation and induces apoptosis by targeting FOXA1. *OncoTargets and therapy* **8**, 2227-2235, doi:10.2147/OTT.S87976 (2015).
89 Tang, J. *et al.* MiR-429 increases the metastatic capability of HCC via regulating classic Wnt pathway rather than epithelial-mesenchymal transition. *Cancer letters* **364**, 33-43, doi:10.1016/j.canlet.2015.04.023 (2015).

90 Huang, J. Y. *et al.* MicroRNA-451: epithelial-mesenchymal transition inhibitor and prognostic biomarker of hepatocelluar carcinoma. *Oncotarget* **6**, 18613-18630, doi:10.18632/oncotarget.4317 (2015).
91 Yu, F., Lu, Z., Chen, B., Dong, P. & Zheng, J. microRNA-150: a promising novel biomarker for hepatitis B virus-related hepatocellular carcinoma. *Diagnostic pathology* **10**, 129, doi:10.1186/s13000-015-0369-y (2015).
92 Li, H., Huang, W. & Luo, R. The microRNA-325 inhibits hepatocellular carcinoma progression by targeting high mobility group box 1. *Diagnostic pathology* **10**, 117, doi:10.1186/s13000-015-0323-z (2015).
93 Su, X. *et al.* An In Vivo Method to Identify microRNA Targets Not Predicted by Computation Algorithms: p21 Targeting by miR-92a in Cancer. *Cancer research* **75**, 2875-2885, doi:10.1158/0008-5472.CAN-14-2218 (2015).
94 Yuan, B., Liang, Y., Wang, D. & Luo, F. MiR-940 inhibits hepatocellular carcinoma growth and correlates with prognosis of hepatocellular carcinoma patients. *Cancer science* **106**, 819-824, doi:10.1111/cas.12688 (2015).
95 Leung, W. K., He, M., Chan, A. W., Law, P. T. & Wong, N. Wnt/beta-Catenin activates MiR-183/96/182 expression in hepatocellular carcinoma that promotes cell invasion. *Cancer letters* **362**, 97-105, doi:10.1016/j.canlet.2015.03.023 (2015).
96 Zhang, J., Chong, C. C., Chen, G. G. & Lai, P. B. A Seven-microRNA Expression Signature Predicts Survival in Hepatocellular Carcinoma. *PloS one* **10**, e0128628, doi:10.1371/journal.pone.0128628 (2015).
97 Tan, Y. L. *et al.* miR-744 is a potential prognostic marker in patients with hepatocellular carcinoma. *Clinics and research in hepatology and gastroenterology* **39**, 359-365, doi:10.1016/j.clinre.2014.09.010 (2015).
98 Huang, C. S. *et al.* Increased expression of miR-21 predicts poor prognosis in patients with hepatocellular carcinoma. *International journal of clinical and experimental pathology* **8**, 7234-7238 (2015).
99 Huang, C. Y. *et al.* miR-128-3p suppresses hepatocellular carcinoma proliferation by regulating PIK3R1 and is correlated with the prognosis of HCC patients. *Oncology reports* **33**, 2889-2898, doi:10.3892/or.2015.3936 (2015).
100 Xu, Y., Bu, X., Dai, C. & Shang, C. High serum microRNA-122 level is independently associated with higher overall survival rate in hepatocellular carcinoma patients. *Tumour biology : the journal of the International Society for Oncodevelopmental Biology and Medicine* **36**, 4773-4776, doi:10.1007/s13277-015-3128-5 (2015).
101 Dou, C. *et al.* MicroRNA-212 suppresses tumor growth of human hepatocellular carcinoma by targeting FOXA1. *Oncotarget* **6**, 13216-13228, doi:10.18632/oncotarget.3916 (2015).
102 Yang, X. *et al.* MicroRNA-200a suppresses metastatic potential of side population cells in human hepatocellular carcinoma by decreasing ZEB2. *Oncotarget* **6**, 7918-7929, doi:10.18632/oncotarget.3486 (2015).
103 Wu, G. *et al.* Low mir-372 expression correlates with poor prognosis and tumor metastasis in hepatocellular carcinoma. *BMC cancer* **15**, 182, doi:10.1186/s12885-015-1214-0 (2015).
104 Liu, F. Y. *et al.* MiR-216b is involved in pathogenesis and progression of hepatocellular carcinoma through HBx-miR-216b-IGF2BP2 signaling pathway. *Cell death & disease* **6**, e1670, doi:10.1038/cddis.2015.46 (2015).
105 Vaira, V. *et al.* MicroRNA-425-3p predicts response to sorafenib therapy in patients with hepatocellular carcinoma. *Liver international : official journal of the International Association for the Study of the Liver* **35**, 1077-1086, doi:10.1111/liv.12636 (2015).
106 Zhou, X. *et al.* miR-625 suppresses tumour migration and invasion by targeting IGF2BP1 in hepatocellular carcinoma. *Oncogene* **34**, 965-977, doi:10.1038/onc.2014.35 (2015).
107 Zheng, F. *et al.* Systemic delivery of microRNA-101 potently inhibits hepatocellular carcinoma in vivo by repressing multiple targets. *PLoS genetics* **11**, e1004873, doi:10.1371/journal.pgen.1004873 (2015).
108 Zhuang, L., Xu, L., Wang, P. & Meng, Z. Serum miR-128-2 serves as a prognostic marker for patients with hepatocellular carcinoma. *PloS one* **10**, e0117274, doi:10.1371/journal.pone.0117274 (2015).

109 Li, L. *et al.* Regulatory MiR-148a-ACVR1/BMP circuit defines a cancer stem cell-like aggressive subtype of hepatocellular carcinoma. *Hepatology* **61**, 574-584, doi:10.1002/hep.27543 (2015).
110 Chen, Z. *et al.* Prognostic significance and anti-proliferation effect of microRNA-365 in hepatocellular carcinoma. *International journal of clinical and experimental pathology* **8**, 1705-1711 (2015).
111 Huang, B., Li, H., Huang, L., Luo, C. & Zhang, Y. Clinical significance of microRNA 138 and cyclin D3 in hepatocellular carcinoma. *The Journal of surgical research* **193**, 718-723, doi:10.1016/j.jss.2014.03.076 (2015).
112 Feng, J. *et al.* miR-200a suppresses cell growth and migration by targeting MACC1 and predicts prognosis in hepatocellular carcinoma. *Oncology reports* **33**, 713-720, doi:10.3892/or.2014.3642 (2015).
113 Zhang, J. G. *et al.* MiR-148b suppresses cell proliferation and invasion in hepatocellular carcinoma by targeting WNT1/beta-catenin pathway. *Scientific reports* **5**, 8087, doi:10.1038/srep08087 (2015).
114 Zhang, Z., Zhang, Y., Sun, X. X., Ma, X. & Chen, Z. N. microRNA-146a inhibits cancer metastasis by downregulating VEGF through dual pathways in hepatocellular carcinoma. *Molecular cancer* **14**, 5, doi:10.1186/1476-4598-14-5 (2015).
115 Kan, H., Guo, W., Huang, Y. & Liu, D. MicroRNA-520g induces epithelial-mesenchymal transition and promotes metastasis of hepatocellular carcinoma by targeting SMAD7. *FEBS letters* **589**, 102-109, doi:10.1016/j.febslet.2014.11.031 (2015).
116 Zhuang, L. P. & Meng, Z. Q. Serum miR-224 reflects stage of hepatocellular carcinoma and predicts survival. *BioMed research international* **2015**, 731781, doi:10.1155/2015/731781 (2015).
117 Song, W. H. *et al.* microRNA-622 acts as a tumor suppressor in hepatocellular carcinoma. *Cancer biology & therapy* **16**, 1754-1763, doi:10.1080/15384047.2015.1095402 (2015).
118 Wang, L. *et al.* Identification of recurrence-related serum microRNAs in hepatocellular carcinoma following hepatectomy. *Cancer biology & therapy* **16**, 1445-1452, doi:10.1080/15384047.2015.1071730 (2015).
119 Tang, D., Sun, B., Yu, H., Yang, Z. & Zhu, L. Tumor-suppressing effect of miR-4458 on human hepatocellular carcinoma. *Cellular physiology and biochemistry : international journal of experimental cellular physiology, biochemistry, and pharmacology* **35**, 1797-1807, doi:10.1159/000373991 (2015).
120 Wang, X. *et al.* Downregulation of MicroRNA-1 is Associated with Poor Prognosis in Hepatocellular Carcinoma. *Clinical laboratory* **61**, 1331-1336 (2015).
121 Peveling-Oberhag, J. *et al.* Feasibility of global miRNA analysis from fine-needle biopsy FFPE material in patients with hepatocellular carcinoma treated with sorafenib. *Clinical science* **128**, 29-37, doi:10.1042/CS20140007 (2015).
122 Xue, T. M. *et al.* Clinicopathological Significance of MicroRNA-20b Expression in Hepatocellular Carcinoma and Regulation of HIF-1alpha and VEGF Effect on Cell Biological Behaviour. *Disease markers* **2015**, 325176, doi:10.1155/2015/325176 (2015).
123 Li, Y. *et al.* Inhibition of the cancer stem cells-like properties by arsenic trioxide, involved in the attenuation of endogenous transforming growth factor beta signal. *Toxicological sciences : an official journal of the Society of Toxicology* **143**, 156-164, doi:10.1093/toxsci/kfu218 (2015).
124 Cai, L. & Cai, X. Up-regulation of miR-9 expression predicate advanced clinicopathological features and poor prognosis in patients with hepatocellular carcinoma. *Diagnostic pathology* **9**, 1000, doi:10.1186/s13000-014-0228-2 (2014).
125 Zhou, H. C. *et al.* Downregulation of microRNA-100 enhances the ICMT-Rac1 signaling and promotes metastasis of hepatocellular carcinoma cells. *Oncotarget* **5**, 12177-12188, doi:10.18632/oncotarget.2601 (2014).
126 Zeng, X. C. *et al.* Downregulation of miR-610 promotes proliferation and tumorigenicity and activates Wnt/beta-catenin signaling in human hepatocellular carcinoma. *Molecular cancer* **13**, 261, doi:10.1186/1476-4598-13-261 (2014).

127 Li, B. K. *et al.* Upregulation of microRNA-106b is associated with poor prognosis in hepatocellular carcinoma. *Diagnostic pathology* **9**, 226, doi:10.1186/s13000-014-0226-4 (2014).
128 Wang, W. Y. *et al.* miR-21 expression predicts prognosis in hepatocellular carcinoma. *Clinics and research in hepatology and gastroenterology* **38**, 715-719, doi:10.1016/j.clinre.2014.07.001 (2014).
129 Song, J. *et al.* MiR-199a regulates cell proliferation and survival by targeting FZD7. *PloS one* **9**, e110074, doi:10.1371/journal.pone.0110074 (2014).
130 Liu, M. *et al.* Association of serum microRNA expression in hepatocellular carcinomas treated with transarterial chemoembolization and patient survival. *PloS one* **9**, e109347, doi:10.1371/journal.pone.0109347 (2014).
131 Mao, K. *et al.* Restoration of miR-193b sensitizes Hepatitis B virus-associated hepatocellular carcinoma to sorafenib. *Cancer letters* **352**, 245-252, doi:10.1016/j.canlet.2014.07.004 (2014).
132 Tsang, F. H. *et al.* Prognostic marker microRNA-125b inhibits tumorigenic properties of hepatocellular carcinoma cells via suppressing tumorigenic molecule eIF5A2. *Digestive diseases and sciences* **59**, 2477-2487, doi:10.1007/s10620-014-3184-5 (2014).
133 Chang, R. M., Yang, H., Fang, F., Xu, J. F. & Yang, L. Y. MicroRNA-331-3p promotes proliferation and metastasis of hepatocellular carcinoma by targeting PH domain and leucine-rich repeat protein phosphatase. *Hepatology* **60**, 1251-1263, doi:10.1002/hep.27221 (2014).
134 Li, B. *et al.* MicroRNA-130a is down-regulated in hepatocellular carcinoma and associates with poor prognosis. *Medical oncology* **31**, 230, doi:10.1007/s12032-014-0230-2 (2014).
135 He, H. *et al.* [Expression of miR-126/miR-126* in hepatocelluar carcinoma and its correlation with clinical outcomes]. *Nan fang yi ke da xue xue bao = Journal of Southern Medical University* **34**, 1493-1497 (2014).
136 Tu, K. *et al.* Prognostic significance of miR-218 in human hepatocellular carcinoma and its role in cell growth. *Oncology reports* **32**, 1571-1577, doi:10.3892/or.2014.3386 (2014).
137 Jiang, J. *et al.* MicroRNA-492 expression promotes the progression of hepatic cancer by targeting PTEN. *Cancer cell international* **14**, 95, doi:10.1186/s12935-014-0095-7 (2014).
138 Parpart, S. *et al.* Modulation of miR-29 expression by alpha-fetoprotein is linked to the hepatocellular carcinoma epigenome. *Hepatology* **60**, 872-883, doi:10.1002/hep.27200 (2014).
139 Meng, F. L., Wang, W. & Jia, W. D. Diagnostic and prognostic significance of serum miR-24-3p in HBV-related hepatocellular carcinoma. *Medical oncology* **31**, 177, doi:10.1007/s12032-014-0177-3 (2014).
140 Sun, Z. *et al.* MicroRNA-1246 enhances migration and invasion through CADM1 in hepatocellular carcinoma. *BMC cancer* **14**, 616, doi:10.1186/1471-2407-14-616 (2014).
141 Liu, Z., Tu, K. & Liu, Q. Effects of microRNA-30a on migration, invasion and prognosis of hepatocellular carcinoma. *FEBS letters* **588**, 3089-3097, doi:10.1016/j.febslet.2014.06.037 (2014).
142 Wang, W. Y. *et al.* High expression of microRNA-130b correlates with poor prognosis of patients with hepatocellular carcinoma. *Diagnostic pathology* **9**, 160, doi:10.1186/s13000-014-0160-5 (2014).
143 Yu, L., Zhang, J., Guo, X., Li, Z. & Zhang, P. MicroRNA-224 upregulation and AKT activation synergistically predict poor prognosis in patients with hepatocellular carcinoma. *Cancer epidemiology* **38**, 408-413, doi:10.1016/j.canep.2014.05.001 (2014).
144 Zhan, M. *et al.* Serum microRNA-210 as a predictive biomarker for treatment response and prognosis in patients with hepatocellular carcinoma undergoing transarterial chemoembolization. *Journal of vascular and interventional radiology : JVIR* **25**, 1279-1287 e1271, doi:10.1016/j.jvir.2014.04.013 (2014).
145 Chai, S. *et al.* Regulatory role of miR-142-3p on the functional hepatic cancer stem cell marker CD133. *Oncotarget* **5**, 5725-5735, doi:10.18632/oncotarget.2167 (2014).
146 Liu, L. L. *et al.* FoxD3-regulated microRNA-137 suppresses tumour growth and metastasis in human hepatocellular carcinoma by targeting AKT2. *Oncotarget* **5**, 5113-5124, doi:10.18632/oncotarget.2089 (2014).

147 Bao, L. *et al.* Correlation between miR-23a and onset of hepatocellular carcinoma. *Clinics and research in hepatology and gastroenterology* **38**, 318-330, doi:10.1016/j.clinre.2013.12.002 (2014).
148 Zhang, Z., Zheng, W. & Hai, J. MicroRNA-148b expression is decreased in hepatocellular carcinoma and associated with prognosis. *Medical oncology* **31**, 984, doi:10.1007/s12032-014-0984-6 (2014).
149 Heo, M. J. *et al.* microRNA-148a dysregulation discriminates poor prognosis of hepatocellular carcinoma in association with USP4 overexpression. *Oncotarget* **5**, 2792-2806, doi:10.18632/oncotarget.1920 (2014).
150 Yang, X. *et al.* MicroRNA-26a suppresses angiogenesis in human hepatocellular carcinoma by targeting hepatocyte growth factor-cMet pathway. *Hepatology* **59**, 1874-1885, doi:10.1002/hep.26941 (2014).
151 Zhang, J. *et al.* MiRNA-99a directly regulates AGO2 through translational repression in hepatocellular carcinoma. *Oncogenesis* **3**, e97, doi:10.1038/oncsis.2014.11 (2014).
152 Li, T. *et al.* Downregulation of microRNA-139 is associated with hepatocellular carcinoma risk and short-term survival. *Oncology reports* **31**, 1699-1706, doi:10.3892/or.2014.3032 (2014).
153 Su, Z. X. *et al.* Upregulation of microRNA-25 associates with prognosis in hepatocellular carcinoma. *Diagnostic pathology* **9**, 47, doi:10.1186/1746-1596-9-47 (2014).
154 Liu, Y. *et al.* MiR-141 suppresses the migration and invasion of HCC cells by targeting Tiam1. *PloS one* **9**, e88393, doi:10.1371/journal.pone.0088393 (2014).
155 Yeh, T. S. *et al.* Expression profile of microRNA-200 family in hepatocellular carcinoma with bile duct tumor thrombus. *Annals of surgery* **259**, 346-354, doi:10.1097/SLA.0000000000000223 (2014).
156 Yang, F. *et al.* MicroRNA-34a targets Bcl-2 and sensitizes human hepatocellular carcinoma cells to sorafenib treatment. *Technology in cancer research & treatment* **13**, 77-86, doi:10.7785/tcrt.2012.500364 (2014).
157 Yang, J. *et al.* Prognostic role of microRNA-221 in various human malignant neoplasms: a meta-analysis of 20 related studies. *PloS one* **9**, e87606, doi:10.1371/journal.pone.0087606 (2014).
158 Liu, Y. X. *et al.* MicroRNA-24 modulates aflatoxin B1-related hepatocellular carcinoma prognosis and tumorigenesis. *BioMed research international* **2014**, 482926, doi:10.1155/2014/482926 (2014).
159 Chen, K. J. *et al.* Reexpression of Let-7g microRNA inhibits the proliferation and migration via K-Ras/HMGA2/snail axis in hepatocellular carcinoma. *BioMed research international* **2014**, 742417, doi:10.1155/2014/742417 (2014).
160 Xu, W. P. *et al.* Perturbation of MicroRNA-370/Lin-28 homolog A/nuclear factor kappa B regulatory circuit contributes to the development of hepatocellular carcinoma. *Hepatology* **58**, 1977-1991, doi:10.1002/hep.26541 (2013).
161 Koberle, V. *et al.* Serum microRNA-1 and microRNA-122 are prognostic markers in patients with hepatocellular carcinoma. *European journal of cancer* **49**, 3442-3449, doi:10.1016/j.ejca.2013.06.002 (2013).
162 Chen, P., Zhao, X. & Ma, L. Downregulation of microRNA-100 correlates with tumor progression and poor prognosis in hepatocellular carcinoma. *Molecular and cellular biochemistry* **383**, 49-58, doi:10.1007/s11010-013-1753-0 (2013).
163 Xiao, F. *et al.* microRNA-200a is an independent prognostic factor of hepatocellular carcinoma and induces cell cycle arrest by targeting CDK6. *Oncology reports* **30**, 2203-2210, doi:10.3892/or.2013.2715 (2013).
164 Wang, J., Li, J., Wang, X., Zheng, C. & Ma, W. Downregulation of microRNA-214 and overexpression of FGFR-1 contribute to hepatocellular carcinoma metastasis. *Biochemical and biophysical research communications* **439**, 47-53, doi:10.1016/j.bbrc.2013.08.032 (2013).
165 Xiao, F. *et al.* MicroRNA-503 inhibits the G1/S transition by downregulating cyclin D3 and E2F3 in hepatocellular carcinoma. *Journal of translational medicine* **11**, 195, doi:10.1186/1479-5876-11-195 (2013).

166 Xia, H., Ooi, L. L. & Hui, K. M. MicroRNA-216a/217-induced epithelial-mesenchymal transition targets PTEN and SMAD7 to promote drug resistance and recurrence of liver cancer. *Hepatology* **58**, 629-641, doi:10.1002/hep.26369 (2013).
167 Wang, R. *et al.* MicroRNA-195 suppresses angiogenesis and metastasis of hepatocellular carcinoma by inhibiting the expression of VEGF, VAV2, and CDC42. *Hepatology* **58**, 642-653, doi:10.1002/hep.26373 (2013).
168 Wang, Y. *et al.* MicroRNA-224 targets SMAD family member 4 to promote cell proliferation and negatively influence patient survival. *PloS one* **8**, e68744, doi:10.1371/journal.pone.0068744 (2013).
169 Yang, H., Fang, F., Chang, R. & Yang, L. MicroRNA-140-5p suppresses tumor growth and metastasis by targeting transforming growth factor beta receptor 1 and fibroblast growth factor 9 in hepatocellular carcinoma. *Hepatology* **58**, 205-217, doi:10.1002/hep.26315 (2013).
170 Yang, X. *et al.* MicroRNA-26a suppresses tumor growth and metastasis of human hepatocellular carcinoma by targeting interleukin-6-Stat3 pathway. *Hepatology* **58**, 158-170, doi:10.1002/hep.26305 (2013).
171 Zhan, M. X. *et al.* [Expression of serum microRNAs (miR-222, miR-181, miR-216) in human hepatocellular carcinoma and its clinical significance]. *Zhonghua yi xue za zhi* **93**, 1830-1832 (2013).
172 Zhi, Q. *et al.* Metastasis-related miR-185 is a potential prognostic biomarker for hepatocellular carcinoma in early stage. *Biomedicine & pharmacotherapy = Biomedecine & pharmacotherapie* **67**, 393-398, doi:10.1016/j.biopha.2013.03.022 (2013).
173 Chen, H. *et al.* Decreased expression of miR-126 correlates with metastatic recurrence of hepatocellular carcinoma. *Clinical & experimental metastasis* **30**, 651-658, doi:10.1007/s10585-013-9569-6 (2013).
174 Law, P. T. *et al.* Deep sequencing of small RNA transcriptome reveals novel non-coding RNAs in hepatocellular carcinoma. *Journal of hepatology* **58**, 1165-1173, doi:10.1016/j.jhep.2013.01.032 (2013).
175 Zheng, J., Dong, P., Gao, S., Wang, N. & Yu, F. High expression of serum miR-17-5p associated with poor prognosis in patients with hepatocellular carcinoma. *Hepato-gastroenterology* **60**, 549-552, doi:10.5754/hge12754 (2013).
176 Fan, M. Q. *et al.* Decrease expression of microRNA-20a promotes cancer cell proliferation and predicts poor survival of hepatocellular carcinoma. *Journal of experimental & clinical cancer research : CR* **32**, 21, doi:10.1186/1756-9966-32-21 (2013).
177 Karakatsanis, A. *et al.* Expression of microRNAs, miR-21, miR-31, miR-122, miR-145, miR-146a, miR-200c, miR-221, miR-222, and miR-223 in patients with hepatocellular carcinoma or intrahepatic cholangiocarcinoma and its prognostic significance. *Molecular carcinogenesis* **52**, 297-303, doi:10.1002/mc.21864 (2013).
178 Zheng, J. J., Yu, F. J., Dong, P. H., Bai, Y. H. & Chen, B. C. [Expression of miRNA-29b and its clinical significances in primary hepatic carcinoma]. *Zhonghua yi xue za zhi* **93**, 888-891 (2013).
179 Han, Z. B. *et al.* [Expression and survival prediction of microRNA-155 in hepatocellular carcinoma after liver transplantation]. *Zhonghua yi xue za zhi* **93**, 884-887 (2013).
180 Gu, H., Guo, X., Zou, L., Zhu, H. & Zhang, J. Upregulation of microRNA-372 associates with tumor progression and prognosis in hepatocellular carcinoma. *Molecular and cellular biochemistry* **375**, 23-30, doi:10.1007/s11010-012-1521-6 (2013).
181 Zhang, Y. *et al.* MicroRNA-101 suppresses SOX9-dependent tumorigenicity and promotes favorable prognosis of human hepatocellular carcinoma. *FEBS letters* **586**, 4362-4370, doi:10.1016/j.febslet.2012.10.053 (2012).
182 Oishi, N. *et al.* Transcriptomic profiling reveals hepatic stem-like gene signatures and interplay of miR-200c and epithelial-mesenchymal transition in intrahepatic cholangiocarcinoma. *Hepatology* **56**, 1792-1803, doi:10.1002/hep.25890 (2012).
183 Li, Q. J. *et al.* MicroRNA-10b promotes migration and invasion through CADM1 in human hepatocellular carcinoma cells. *Tumour biology : the journal of the International Society for Oncodevelopmental Biology and Medicine* **33**, 1455-1465, doi:10.1007/s13277-012-0396-1 (2012).

184 Chen, H. Y. *et al.* miR-203 expression predicts outcome after liver transplantation for hepatocellular carcinoma in cirrhotic liver. *Medical oncology* **29**, 1859-1865, doi:10.1007/s12032-011-0031-9 (2012).

185 Han, Z. B. *et al.* Identification of recurrence-related microRNAs in hepatocellular carcinoma following liver transplantation. *Molecular oncology* **6**, 445-457, doi:10.1016/j.molonc.2012.04.001 (2012).

186 Law, P. T. *et al.* MiR-145 modulates multiple components of the insulin-like growth factor pathway in hepatocellular carcinoma. *Carcinogenesis* **33**, 1134-1141, doi:10.1093/carcin/bgs130 (2012).

187 Chen, L., Jiang, M., Yuan, W. & Tang, H. miR-17-5p as a novel prognostic marker for hepatocellular carcinoma. *Journal of investigative surgery : the official journal of the Academy of Surgical Research* **25**, 156-161, doi:10.3109/08941939.2011.618523 (2012).

188 Augello, C. *et al.* MicroRNA profiling of hepatocarcinogenesis identifies C19MC cluster as a novel prognostic biomarker in hepatocellular carcinoma. *Liver international : official journal of the International Association for the Study of the Liver* **32**, 772-782, doi:10.1111/j.1478-3231.2012.02795.x (2012).

189 Huang, N. *et al.* MiR-219-5p inhibits hepatocellular carcinoma cell proliferation by targeting glypican-3. *FEBS letters* **586**, 884-891, doi:10.1016/j.febslet.2012.02.017 (2012).

190 Liu, S. *et al.* MicroRNA-135a contributes to the development of portal vein tumor thrombus by promoting metastasis in hepatocellular carcinoma. *Journal of hepatology* **56**, 389-396, doi:10.1016/j.jhep.2011.08.008 (2012).

191 Han, Z. B. *et al.* Up-regulation of microRNA-155 promotes cancer cell invasion and predicts poor survival of hepatocellular carcinoma following liver transplantation. *Journal of cancer research and clinical oncology* **138**, 153-161, doi:10.1007/s00432-011-1076-z (2012).

192 Zhu, H. T. *et al.* MicroRNA-29a-5p is a novel predictor for early recurrence of hepatitis B virus-related hepatocellular carcinoma after surgical resection. *PloS one* **7**, e52393, doi:10.1371/journal.pone.0052393 (2012).

193 Xia, H., Ooi, L. L. & Hui, K. M. MiR-214 targets beta-catenin pathway to suppress invasion, stem-like traits and recurrence of human hepatocellular carcinoma. *PloS one* **7**, e44206, doi:10.1371/journal.pone.0044206 (2012).

194 Huang, Y. H. *et al.* Identification of postoperative prognostic microRNA predictors in hepatocellular carcinoma. *PloS one* **7**, e37188, doi:10.1371/journal.pone.0037188 (2012).

195 Wang, C. *et al.* Underexpressed microRNA-199b-5p targets hypoxia-inducible factor-1alpha in hepatocellular carcinoma and predicts prognosis of hepatocellular carcinoma patients. *Journal of gastroenterology and hepatology* **26**, 1630-1637, doi:10.1111/j.1440-1746.2011.06758.x (2011).

196 Li, D. *et al.* MicroRNA-99a inhibits hepatocellular carcinoma growth and correlates with prognosis of patients with hepatocellular carcinoma. *The Journal of biological chemistry* **286**, 36677-36685, doi:10.1074/jbc.M111.270561 (2011).

197 Yoon, S. O. *et al.* Deregulated expression of microRNA-221 with the potential for prognostic biomarkers in surgically resected hepatocellular carcinoma. *Human pathology* **42**, 1391-1400, doi:10.1016/j.humpath.2010.12.010 (2011).

198 Li, J., Wang, Y., Yu, W., Chen, J. & Luo, J. Expression of serum miR-221 in human hepatocellular carcinoma and its prognostic significance. *Biochemical and biophysical research communications* **406**, 70-73, doi:10.1016/j.bbrc.2011.01.111 (2011).

199 Hou, J. *et al.* Identification of miRNomes in human liver and hepatocellular carcinoma reveals miR-199a/b-3p as therapeutic target for hepatocellular carcinoma. *Cancer cell* **19**, 232-243, doi:10.1016/j.ccr.2011.01.001 (2011).

200 Wong, C. C. *et al.* The microRNA miR-139 suppresses metastasis and progression of hepatocellular carcinoma by down-regulating Rho-kinase 2. *Gastroenterology* **140**, 322-331, doi:10.1053/j.gastro.2010.10.006 (2011).

201 Tomimaru, Y. *et al.* MicroRNA-21 induces resistance to the anti-tumour effect of interferon-alpha/5-fluorouracil in hepatocellular carcinoma cells. *British journal of cancer* **103**, 1617-1626, doi:10.1038/sj.bjc.6605958 (2010).

202 Zhang, J. *et al.* microRNA-22, downregulated in hepatocellular carcinoma and correlated with prognosis, suppresses cell proliferation and tumourigenicity. *British journal of cancer* **103**, 1215-1220, doi:10.1038/sj.bjc.6605895 (2010).
203 Burchard, J. *et al.* microRNA-122 as a regulator of mitochondrial metabolic gene network in hepatocellular carcinoma. *Molecular systems biology* **6**, 402, doi:10.1038/msb.2010.58 (2010).
204 Ji, J. *et al.* Let-7g targets collagen type I alpha2 and inhibits cell migration in hepatocellular carcinoma. *Journal of hepatology* **52**, 690-697, doi:10.1016/j.jhep.2009.12.025 (2010).
205 Xiong, Y. *et al.* Effects of microRNA-29 on apoptosis, tumorigenicity, and prognosis of hepatocellular carcinoma. *Hepatology* **51**, 836-845, doi:10.1002/hep.23380 (2010).
206 Wong, Q. W. *et al.* MiR-222 overexpression confers cell migratory advantages in hepatocellular carcinoma through enhancing AKT signaling. *Clinical cancer research : an official journal of the American Association for Cancer Research* **16**, 867-875, doi:10.1158/1078-0432.CCR-09-1840 (2010).
207 Ji, J. *et al.* MicroRNA expression, survival, and response to interferon in liver cancer. *The New England journal of medicine* **361**, 1437-1447, doi:10.1056/NEJMoa0901282 (2009).
208 Coulouarn, C., Factor, V. M., Andersen, J. B., Durkin, M. E. & Thorgeirsson, S. S. Loss of miR-122 expression in liver cancer correlates with suppression of the hepatic phenotype and gain of metastatic properties. *Oncogene* **28**, 3526-3536, doi:10.1038/onc.2009.211 (2009).
209 Gramantieri, L. *et al.* MicroRNA-221 targets Bmf in hepatocellular carcinoma and correlates with tumor multifocality. *Clinical cancer research : an official journal of the American Association for Cancer Research* **15**, 5073-5081, doi:10.1158/1078-0432.CCR-09-0092 (2009).
210 Fornari, F. *et al.* MiR-122/cyclin G1 interaction modulates p53 activity and affects doxorubicin sensitivity of human hepatocarcinoma cells. *Cancer research* **69**, 5761-5767, doi:10.1158/0008-5472.CAN-08-4797 (2009).
211 Li, W. *et al.* Diagnostic and prognostic implications of microRNAs in human hepatocellular carcinoma. *International journal of cancer* **123**, 1616-1622, doi:10.1002/ijc.23693 (2008).
212 Murakami, Y. *et al.* The expression level of miR-18b in hepatocellular carcinoma is associated with the grade of malignancy and prognosis. *BMC cancer* **13**, 99, doi:10.1186/1471-2407-13-99 (2013).
213 Cui, L., Hu, Y., Bai, B. & Zhang, S. Serum miR-335 Level is Associated with the Treatment Response to Trans-Arterial Chemoembolization and Prognosis in Patients with Hepatocellular Carcinoma. *Cellular physiology and biochemistry : international journal of experimental cellular physiology, biochemistry, and pharmacology* **37**, 276-283, doi:10.1159/000430352 (2015).
214 Sato, F. *et al.* MicroRNA profile predicts recurrence after resection in patients with hepatocellular carcinoma within the Milan Criteria. *PloS one* **6**, e16435, doi:10.1371/journal.pone.0016435 (2011).

Supplemental Table 3. List of miRNAs significant in overall survival analysis using the TCGA **(A)** and overall survival and relapse-free survival analysis using the GSE31384 **(B and C)** dataset.

A)

miRNA	HR	*p*-value (univariate analysis)	*p*-value (multivariate analysis)*	*p*-value (multivariate analysis)**
hsa-miR-149	0.36	5.19E-09	2.24E-06	2.26E-03
hsa-miR-139	2.48	4.50E-07	6.02E-04	9.45E-01
hsa-miR-3677	0.41	1.24E-06	2.39E-04	8.59E-01
hsa-miR-550a	0.44	2.45E-06	5.95E-04	2.83E-01
hsa-miR-212	0.45	7.53E-06	1.92E-04	6.93E-01
hsa-miR-421	0.48	2.66E-05	5.20E-03	8.21E-01
hsa-miR-3607	2.09	4.29E-05	5.42E-03	4.94E-04
hsa-miR-326	0.5	1.00E-04	3.04E-04	1.39E-01
hsa-miR-940	0.49	1.00E-04	2.86E-04	3.84E-01
hsa-miR-100	1.96	2.00E-04	3.74E-03	9.13E-01
hsa-miR-215	0.53	2.00E-04	1.03E-02	1.13E-03
hsa-miR-454	0.52	2.00E-04	6.16E-03	1.95E-01
hsa-miR-301a	0.53	3.00E-04	1.52E-03	3.43E-01
hsa-miR-330	0.52	3.00E-04	4.55E-03	2.46E-01
hsa-miR-22	1.92	3.00E-04	8.52E-04	3.90E-01
hsa-miR-491	0.54	5.00E-04	4.67E-03	1.04E-01
hsa-miR-9	0.54	6.00E-04	2.52E-03	5.10E-01
hsa-miR-15b	0.55	6.00E-04	1.08E-02	8.17E-01
hsa-miR-25	0.54	6.00E-04	3.82E-03	2.39E-01
hsa-miR-34a	1.78	1.10E-03	3.27E-03	6.92E-02
hsa-miR-126	1.76	1.20E-03	8.52E-02	2.76E-01
hsa-miR-132	0.58	1.60E-03	7.67E-03	3.84E-02
hsa-miR-301b	0.56	1.80E-03	6.71E-04	6.47E-01
hsa-miR-24	0.57	2.10E-03	1.09E-02	4.16E-01
hsa-miR-148a	1.74	2.30E-03	1.12E-02	2.50E-02
hsa-miR-194	1.72	2.50E-03	3.67E-02	4.70E-01
hsa-miR-222	0.54	2.80E-03	6.00E-03	3.57E-01
hsa-miR-101	1.68	3.30E-03	3.88E-02	7.78E-01
hsa-miR-125b	1.85	4.30E-03	1.65E-02	5.67E-01
hsa-miR-744	0.58	5.00E-03	7.20E-04	4.09E-01
hsa-miR-141	0.61	5.70E-03	3.87E-02	4.71E-01
hsa-miR-33a	0.6	5.70E-03	5.29E-03	9.39E-01
hsa-miR-33b	0.6	6.20E-03	1.34E-02	9.65E-01
hsa-miR-99a	1.64	6.20E-03	5.84E-02	7.15E-01
hsa-miR-21	0.61	6.80E-03	7.93E-02	3.17E-01
hsa-miR-20b	1.66	6.80E-03	9.26E-02	7.60E-02
hsa-miR-429	0.62	6.90E-03	1.33E-02	3.27E-01
hsa-miR-1	1.67	7.20E-03	6.78E-03	1.19E-02
hsa-miR-23a	0.61	7.60E-03	8.88E-02	1.74E-01
hsa-miR-186	0.63	9.90E-03	3.79E-02	2.99E-01
hsa-miR-10b	0.63	1.15E-02	8.67E-03	1.70E-02
hsa-miR-182	0.64	1.30E-02	3.32E-01	7.48E-01
hsa-miR-195	1.54	1.43E-02	6.35E-02	4.28E-02
hsa-miR-200c	0.65	1.58E-02	5.82E-02	8.63E-01

hsa-miR-106b	0.59	1.59E-02	6.20E-02	1.02E-01
hsa-miR-122	1.56	1.65E-02	2.83E-01	1.23E-01
hsa-miR-221	0.66	1.72E-02	6.73E-03	3.86E-02
hsa-miR-183	0.67	2.60E-02	2.67E-01	6.75E-01
hsa-miR-204	1.47	3.09E-02	2.23E-01	5.28E-01
hsa-miR-145	1.46	3.21E-02	3.44E-02	2.35E-01
hsa-miR-203a	1.58	3.57E-02	2.42E-01	2.70E-01
hsa-miR-29c	1.45	4.13E-02	6.97E-02	9.58E-01
hsa-miR-223	0.68	4.27E-02	1.07E-01	3.54E-01
hsa-let-7g	1.44	4.55E-02	1.06E-01	3.90E-01
hsa-miR-135a	0.7	4.71E-02	1.01E-02	1.52E-02

B)

**miRNA**	**HR**	***p*-value (univariate analysis)**	**p-value (multivariate analysis)****
hsa-miR-146b-3p	3.05	7.76E-07	6.94E-02
hsa-miR-584	0.33	1.38E-06	
hsa-miR-612	0.37	1.47E-05	4.53E-01
hsa-miR-28-5p	2.69	1.58E-05	
hsa-miR-139-5p	2.87	1.85E-05	
hsa-miR-486-3p	2.6	3.72E-05	4.41E-03
hsa-miR-29c	2.56	5.63E-05	
hsa-miR-34b	3.46	6.04E-05	
hsa-miR-496	3.33	6.23E-05	4.63E-02
hsa-miR-126	3	9.76E-05	
hsa-miR-24	2.42	1.08E-04	9.37E-01
hsa-miR-330-3p	0.41	1.15E-04	1.11E-01
hsa-miR-675	0.4	1.17E-04	
hsa-miR-105	2.53	1.22E-04	5.41E-02
hsa-miR-489	0.41	1.25E-04	
hsa-miR-31	2.42	1.49E-04	4.21E-01
hsa-miR-106b	2.93	1.74E-04	
hsa-miR-99a	0.42	1.76E-04	
hsa-miR-15b	2.79	2.17E-04	3.20E-01
hsa-miR-548b-3p	0.38	2.42E-04	
hsa-miR-185	2.3	2.88E-04	1.48E-01
hsa-miR-302d	2.28	3.41E-04	7.06E-01
hsa-miR-221	0.44	3.75E-04	9.95E-01
hsa-miR-30a	2.31	3.86E-04	3.35E-01
hsa-miR-92a	0.44	4.41E-04	9.59E-01
hsa-miR-301b	2.25	4.42E-04	
hsa-miR-661	0.43	4.52E-04	
hsa-miR-940	0.44	4.60E-04	7.99E-01
hsa-miR-509-3p	0.41	6.98E-04	
hsa-miR-769-5p	0.45	7.54E-04	6.95E-02
hsa-miR-625	0.36	7.84E-04	
hsa-miR-122	2.22	8.10E-04	
hsa-miR-182	2.18	8.57E-04	
hsa-miR-137	0.46	8.69E-04	

hsa-miR-29a	0.33	1.00E-03	1.43E-02
hsa-miR-181b	2.14	1.01E-03	2.48E-01
hsa-miR-155	2.15	1.02E-03	3.68E-01
hsa-miR-503	0.46	1.02E-03	5.99E-01
hsa-miR-9	0.47	1.14E-03	
hsa-miR-608	0.47	1.24E-03	4.66E-01
hsa-miR-421	2.1	1.54E-03	
hsa-miR-325	0.38	1.66E-03	
hsa-miR-522	0.48	1.93E-03	1.04E-01
hsa-miR-142-3p	2.1	2.03E-03	
hsa-miR-638	0.48	2.18E-03	
hsa-miR-29b	2.05	2.26E-03	
hsa-miR-876-5p	0.48	2.30E-03	8.43E-01
hsa-miR-520h	0.44	2.39E-03	5.93E-01
hsa-miR-22	2	3.14E-03	6.72E-01
hsa-miR-10b	2	3.20E-03	2.20E-02
hsa-miR-219-5p	2.3	3.33E-03	
hsa-miR-222	0.5	3.91E-03	7.81E-01
hsa-miR-132	1.99	4.01E-03	
hsa-miR-492	0.51	4.17E-03	
hsa-miR-339-5p	0.52	4.78E-03	5.12E-01
hsa-miR-141	1.95	5.45E-03	
hsa-miR-886-5p	1.92	5.84E-03	8.93E-01
hsa-miR-183	1.89	6.13E-03	
hsa-miR-100	0.52	6.66E-03	1.93E-01
hsa-miR-622	0.54	8.53E-03	5.55E-01
hsa-miR-601	0.44	8.58E-03	5.15E-02
hsa-miR-26a	0.55	8.83E-03	2.42E-01
hsa-miR-326	0.55	9.79E-03	
hsa-miR-138	1.85	1.12E-02	1.04E-01
hsa-miR-203	0.54	1.18E-02	4.24E-01
hsa-miR-216b	1.81	1.19E-02	7.96E-01
hsa-miR-331-3p	0.56	1.19E-02	
hsa-miR-96	0.55	1.23E-02	
hsa-miR-25	0.49	1.24E-02	1.62E-02
hsa-miR-610	0.56	1.27E-02	4.54E-01
hsa-miR-148a	0.55	1.45E-02	
hsa-miR-186	0.55	1.57E-02	4.23E-01
hsa-miR-451	1.7	2.41E-02	6.65E-01
hsa-miR-135a	1.72	2.57E-02	
hsa-miR-21	1.79	2.97E-02	9.07E-01
hsa-miR-218	1.65	3.49E-02	
hsa-miR-634	0.52	3.62E-02	3.51E-01
hsa-miR-630	0.6	3.75E-02	1.93E-02
hsa-miR-590-3p	0.61	3.84E-02	3.33E-01
hsa-miR-145	1.61	4.41E-02	
hsa-miR-140-5p	1.6	4.70E-02	3.92E-02
hsa-miR-212	0.62	4.76E-02	9.30E-01
hsa-miR-383	1.59	4.87E-02	
hsa-miR-195	1.53	7.30E-02	3.04E-02

C)

**miRNA**	**HR**	***p*-value (univariate analysis)**	**p-value (multivariate analysis)****
hsa-miR-126	2.69	4.9E-05	
hsa-miR-122	2.3	5.21E-05	
hsa-miR-106b	2.23	5.56E-05	
hsa-miR-195	1.93	0.000806	0.001688
hsa-miR-30a	1.92	0.000919	
hsa-miR-7	0.44	0.000988	
hsa-miR-139-5p	2.1	0.002976	
hsa-miR-486-3p	1.83	0.003682	0.166657
hsa-miR-142-3p	1.82	0.003897	
hsa-miR-29b	1.75	0.004775	
hsa-miR-378	1.73	0.005122	
hsa-miR-99a	0.56	0.005264	
hsa-miR-381	0.6	0.009009	
hsa-miR-92a	0.6	0.016594	0.01427
hsa-miR-24	1.61	0.018346	0.696488
hsa-miR-19b	1.78	0.019558	
hsa-miR-630	0.62	0.022973	
hsa-miR-26a	0.64	0.023252	0.457265
hsa-miR-103	1.54	0.02937	
hsa-miR-221	0.65	0.030609	0.660817
hsa-miR-9	0.64	0.032165	
hsa-miR-15b	1.54	0.039052	0.245006
hsa-miR-182	1.5	0.040979	
hsa-miR-451	1.51	0.042946	0.52744
hsa-miR-22	1.52	0.044638	0.672517

*Multivariate analysis using stage and gender.
**Multivariate analysis using all significant miRNAs.

Supplemental Table 4. List of miRNAs significant in the differential expression analysis comparing normal and tumor tissue.

miRNA	Dataset	*p*-value	log_2 fold change
hsa-miR-222	GSE6857	1.08E-38	1.01
hsa-miR-21	GSE6857	2.41E-38	1.57
hsa-miR-34a	GSE6857	4.53E-36	0.76
hsa-miR-182	GSE6857	1.48E-35	0.58
hsa-miR-221	GSE6857	2.18E-34	1.27
hsa-miR-140	GSE6857	6.18E-31	0.72
hsa-miR-372	GSE6857	1.18E-29	0.52
hsa-miR-183	GSE6857	1.14E-25	0.45
hsa-miR-302d	GSE6857	1.95E-24	0.60
hsa-miR-15a	GSE6857	1.01E-23	0.68
hsa-miR-135a	GSE6857	1.58E-23	0.73
hsa-miR-130a	TCGA	5.59E-22	-1.97
hsa-miR-139	TCGA	2.57E-21	-2.26
hsa-miR-424	TCGA	1.03E-19	-2.10
hsa-miR-10b	TCGA	1.19E-19	3.46
hsa-miR-221	GSE10694	1.32E-19	2.27
hsa-miR-141	GSE6857	1.36E-19	0.39
hsa-miR-1	GSE6857	1.33E-17	0.41
hsa-miR-101	TCGA	1.16E-16	-1.24
hsa-miR-222	GSE10694	1.25E-16	2.03
hsa-miR-370	GSE6857	4.29E-16	0.29
hsa-miR-330	GSE6857	2.10E-15	0.34
hsa-miR-107	GSE6857	6.75E-15	0.67
hsa-miR-199a	GSE6857	6.09E-14	-0.55
hsa-miR-675	TCGA	6.65E-14	-3.39
hsa-miR-224	GSE10694	1.12E-13	1.30
hsa-miR-137	GSE6857	1.39E-13	0.65
hsa-miR-199a	TCGA	1.78E-13	-2.36
hsa-miR-199b	TCGA	2.47E-13	-2.34
hsa-miR-106b	GSE10694	2.63E-12	1.58
hsa-miR-3677	TCGA	3.68E-12	1.80
hsa-miR-125b	GSE6857	1.44E-11	0.55
hsa-miR-15b	GSE10694	1.94E-11	1.43
hsa-miR-183	TCGA	2.51E-11	2.68
hsa-miR-217	GSE6857	3.31E-11	0.63
hsa-miR-145	TCGA	3.44E-11	-1.35
hsa-miR-181b	GSE6857	3.62E-11	0.37
hsa-miR-422a	GSE10694	4.67E-11	-0.76
hsa-miR-186	GSE6857	9.04E-11	0.29
hsa-miR-21	TCGA	1.25E-10	1.18
hsa-miR-25	GSE10694	1.61E-10	1.36
hsa-miR-125b	TCGA	2.07E-10	-1.36
hsa-miR-223	TCGA	2.13E-10	-1.31
hsa-miR-218	GSE6857	3.53E-10	0.29
hsa-miR-210	GSE6857	6.14E-10	0.46
hsa-miR-99a	TCGA	6.79E-10	-1.76
hsa-miR-210	GSE10694	8.06E-10	1.19

hsa-miR-101	GSE10694	2.49E-09	-1.38
hsa-miR-221	TCGA	2.56E-09	1.06
hsa-miR-99a	GSE10694	2.70E-09	-1.32
hsa-miR-125b	GSE10694	2.96E-09	-1.29
hsa-miR-33b	TCGA	3.71E-09	-1.57
hsa-miR-100	GSE10694	4.98E-09	-1.17
hsa-miR-182	TCGA	6.88E-09	2.17
hsa-miR-30c	TCGA	1.24E-08	-0.81
hsa-miR-215	GSE6857	1.31E-08	-0.56
hsa-miR-497	TCGA	2.13E-08	-1.27
hsa-miR-421	TCGA	3.09E-08	1.15
hsa-miR-214	TCGA	3.69E-08	-2.52
hsa-miR-381	TCGA	3.76E-08	-1.27
hsa-miR-107	GSE10694	4.08E-08	0.91
hsa-miR-25	TCGA	4.43E-08	0.65
hsa-miR-195	TCGA	5.45E-08	-1.26
hsa-miR-455	TCGA	8.36E-08	-1.22
hsa-miR-185	GSE6857	1.12E-07	0.28
hsa-miR-27b	TCGA	1.40E-07	-0.69
hsa-miR-203	GSE6857	1.62E-07	0.24
hsa-miR-204	GSE6857	1.80E-07	0.30
hsa-miR-106b	TCGA	1.91E-07	0.54
hsa-miR-106b	GSE6857	2.24E-07	0.45
hsa-miR-3607	TCGA	2.34E-07	-1.85
hsa-miR-30a	TCGA	2.70E-07	-0.90
hsa-miR-148	GSE6857	2.89E-07	-0.51
hsa-miR-103	GSE10694	2.91E-07	0.93
hsa-miR-185	GSE10694	3.54E-07	0.90
hsa-miR-200c	GSE6857	5.65E-07	0.20
hsa-miR-199a	GSE10694	8.26E-07	-1.31
hsa-miR-101	GSE6857	9.11E-07	0.24
hsa-miR-105	GSE6857	9.15E-07	0.36
hsa-miR-30b	TCGA	1.00E-06	-0.71
hsa-miR-224	TCGA	1.24E-06	1.87
hsa-miR-370	TCGA	1.35E-06	-1.22
hsa-miR-130a	GSE10694	1.58E-06	-0.89
hsa-miR-22	TCGA	1.60E-06	-0.58
hsa-miR-29c	TCGA	1.61E-06	-1.11
hsa-miR-330	TCGA	1.88E-06	0.79
hsa-miR-34a	TCGA	2.06E-06	0.91
hsa-miR-503	TCGA	2.12E-06	-0.89
hsa-miR-125a	TCGA	2.57E-06	-0.87
hsa-miR-212	GSE6857	2.59E-06	0.31
hsa-miR-1468	TCGA	3.01E-06	-0.93
hsa-miR-200b	TCGA	3.03E-06	-2.04
hsa-miR-125a	GSE6857	3.10E-06	-0.32
hsa-miR-150	TCGA	3.84E-06	-1.38
hsa-miR-148b	GSE10694	4.02E-06	0.40
hsa-miR-214	GSE6857	4.02E-06	-0.39
hsa-miR-520f	GSE10694	4.04E-06	-0.59
hsa-miR-200a	TCGA	4.51E-06	-2.00
hsa-miR-26a	GSE6857	5.70E-06	-0.38
hsa-miR-222	TCGA	5.81E-06	0.87
hsa-miR-150	GSE6857	6.23E-06	-0.40
hsa-miR-17-5p	GSE10694	7.73E-06	0.88

hsa-miR-148a	GSE10694	1.15E-05	-0.70
hsa-miR-194	GSE6857	1.74E-05	0.32
hsa-miR-126	TCGA	1.82E-05	-0.66
hsa-miR-429	TCGA	2.04E-05	-4.02
hsa-miR-494	TCGA	2.32E-05	-3.29
hsa-miR-203a	TCGA	2.70E-05	-1.41
hsa-miR-155	GSE6857	3.00E-05	0.43
hsa-miR-375	TCGA	3.46E-05	-1.81
hsa-miR-26a	TCGA	3.67E-05	-0.51
hsa-miR-9	TCGA	4.79E-05	1.69
hsa-miR-326	TCGA	4.91E-05	-0.79
hsa-miR-29c	GSE10694	5.50E-05	-1.00
hsa-miR-149	GSE6857	5.54E-05	0.21
hsa-miR-195	GSE10694	6.43E-05	-0.90
hsa-miR-98	GSE10694	6.63E-05	0.55
hsa-miR-145	GSE6857	6.92E-05	-0.40
hsa-miR-148b	TCGA	7.33E-05	0.39
hsa-miR-23b	TCGA	8.79E-05	-0.42
hsa-miR-148a	TCGA	1.18E-04	-0.66
hsa-miR-34a	GSE10694	1.39E-04	0.69
hsa-miR-30d	TCGA	1.48E-04	0.49
hsa-miR-223	GSE6857	2.13E-04	-0.41
hsa-miR-199b	GSE6857	3.63E-04	-0.22
hsa-miR-122	TCGA	4.78E-04	-1.29
hsa-miR-20a	GSE10694	6.11E-04	0.70
hsa-miR-140	TCGA	6.39E-04	0.37
hsa-miR-424	GSE10694	6.65E-04	-0.68
hsa-miR-132	TCGA	8.45E-04	0.48
hsa-miR-29b	TCGA	1.06E-03	-0.74
hsa-miR-18a	GSE10694	1.10E-03	0.84
hsa-miR-744	TCGA	1.22E-03	-0.43
hsa-miR-192	TCGA	1.37E-03	-0.69
hsa-miR-146a	TCGA	1.84E-03	-0.81
hsa-miR-100	TCGA	1.92E-03	-0.89
hsa-miR-497	GSE10694	2.33E-03	-0.45
hsa-miR-210	TCGA	2.52E-03	-0.79
hsa-miR-33a	TCGA	3.32E-03	-0.56
hsa-miR-141	TCGA	3.91E-03	-1.56
hsa-miR-99b	GSE10694	4.54E-03	0.45
hsa-miR-130b	GSE10694	4.59E-03	0.36
hsa-miR-451	GSE10694	5.61E-03	-0.85
hsa-miR-155	GSE10694	5.73E-03	-0.57
hsa-miR-496	TCGA	5.74E-03	-1.30
hsa-miR-100	GSE6857	5.95E-03	-0.23
hsa-miR-185	TCGA	5.97E-03	0.28
hsa-miR-126	GSE6857	6.75E-03	0.23
hsa-miR-30c	GSE10694	6.84E-03	-0.34
hsa-miR-22	GSE10694	8.05E-03	-0.62
hsa-miR-20b	GSE10694	8.07E-03	0.52
hsa-miR-940	TCGA	8.63E-03	0.95
hsa-miR-432	TCGA	8.92E-03	-0.65
hsa-miR-301b	TCGA	9.25E-03	2.01
hsa-miR-550a	TCGA	9.64E-03	0.39
hsa-miR-214	GSE10694	1.01E-02	-0.55
hsa-miR-126	GSE10694	1.01E-02	-0.39

hsa-miR-30d	GSE10694	1.11E-02	0.49
hsa-miR-29b	GSE6857	1.80E-02	0.16
hsa-miR-192	GSE6857	1.89E-02	-0.22
hsa-miR-145	GSE10694	2.04E-02	-0.42
hsa-let-7g	TCGA	2.08E-02	-0.30
hsa-miR-224	GSE6857	2.12E-02	-0.17
hsa-miR-103	GSE6857	2.15E-02	0.20
hsa-miR-19b	TCGA	2.15E-02	-0.38
hsa-miR-7	TCGA	2.41E-02	-0.36
hsa-miR-325	GSE6857	2.59E-02	-0.25
hsa-miR-96	TCGA	2.63E-02	1.76
hsa-miR-27b	GSE10694	2.69E-02	-0.27
hsa-miR-29a	TCGA	3.02E-02	-0.31
hsa-miR-194	TCGA	3.03E-02	-0.43
hsa-miR-218	TCGA	3.17E-02	-0.50
hsa-miR-98	TCGA	3.68E-02	0.30
hsa-miR-200b	GSE6857	3.70E-02	0.09
hsa-miR-19a	GSE10694	3.70E-02	0.32
hsa-miR-135a	TCGA	4.00E-02	2.46
hsa-miR-139	GSE6857	4.05E-02	-0.11
hsa-miR-20b	TCGA	4.07E-02	-0.49
hsa-miR-125a	GSE10694	4.77E-02	-0.45
hsa-miR-181b	TCGA	4.87E-02	0.31

Supplemental Figure 1.A-F

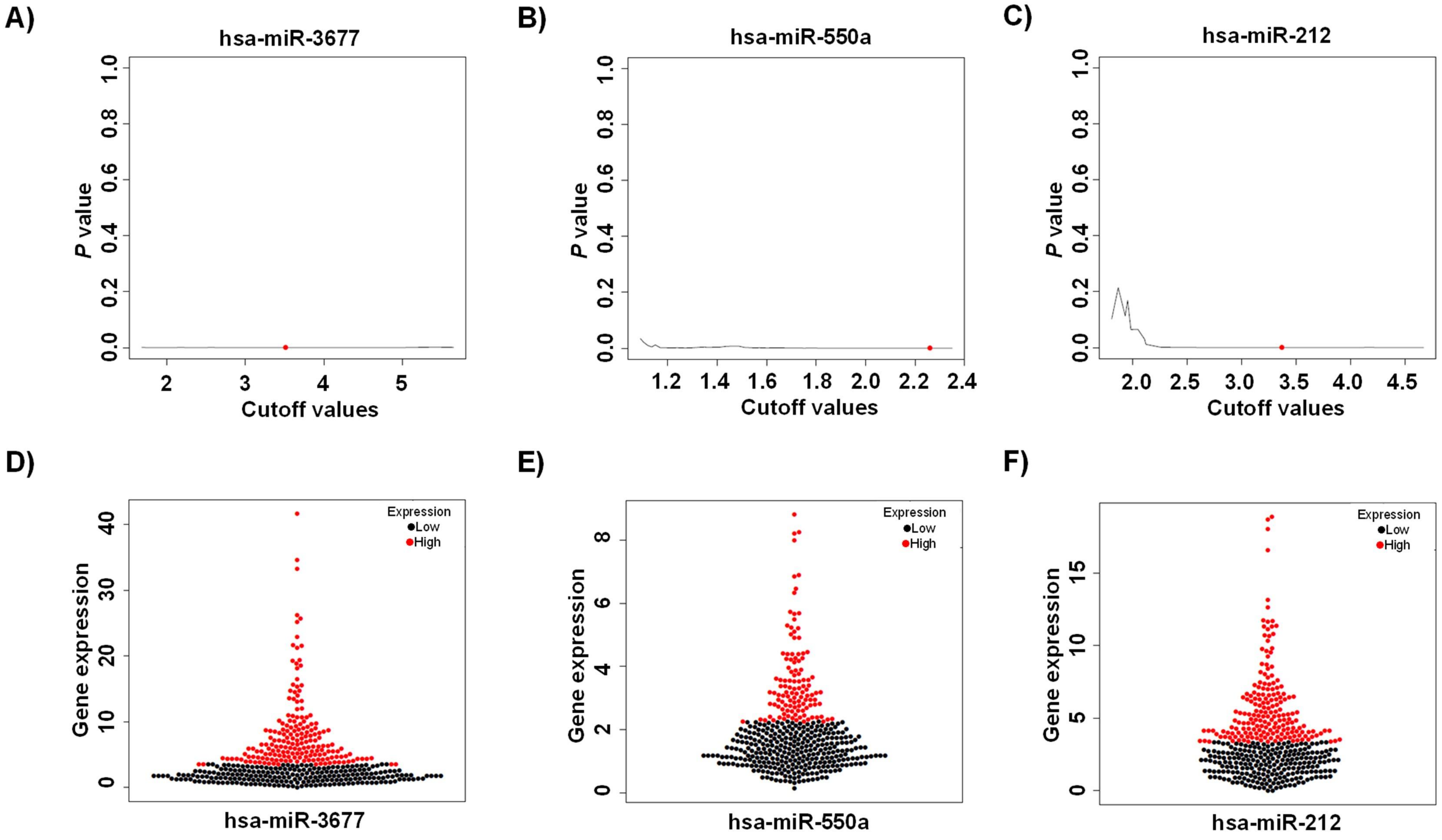

Supplemental Figure 2.A-C

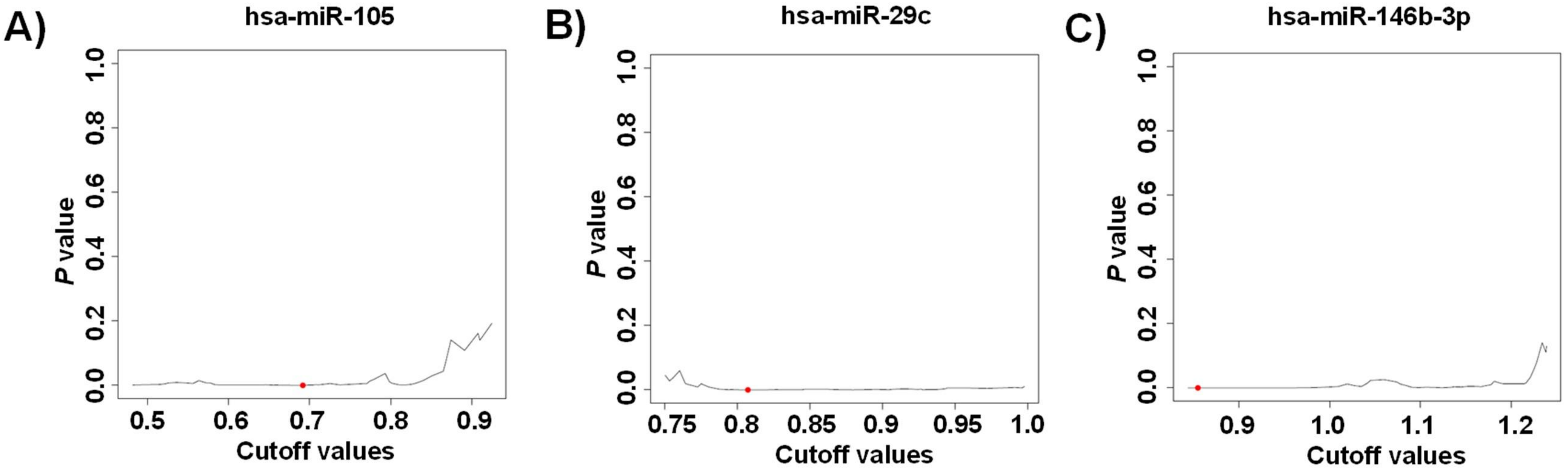

Supplement: Supplementary file 2 — Supplementary Information [file 41598_2018_27521_MOESM2_ESM.pdf]
